# Health-Related Quality of Life, Psychosocial Distress and Unmet Needs in Older Patients With Head and Neck Cancer

**DOI:** 10.3389/fonc.2022.834068

**Published:** 2022-02-15

**Authors:** Lachlan McDowell, Danny Rischin, Karla Gough, Christina Henson

**Affiliations:** ^1^ Department of Radiation Oncology, Peter MacCallum Cancer Centre, Melbourne, VIC, Australia; ^2^ Sir Peter MacCallum Department of Oncology, The University of Melbourne, Melbourne, VIC, Australia; ^3^ Department of Medical Oncology, Peter MacCallum Cancer Centre, Melbourne, VIC, Australia; ^4^ Department of Health Services Research, Peter MacCallum Cancer Centre, Melbourne, VIC, Australia; ^5^ Department of Nursing, Faculty of Medicine, Dentistry and Health Sciences, The University of Melbourne, Carlton, VIC, Australia; ^6^ Department of Radiation Oncology, Stephenson Cancer Center, University of Oklahoma, Oklahoma City, OK, United States

**Keywords:** radiation therapy, head neck cancer, surgery, chemotherapy, quality of life, psychosocial distress, sleep, unmet need

## Abstract

Head and neck squamous cell carcinoma (HNSCC) is the most common cancer involving the mucosal surfaces of the head and neck and is associated with a number of etiological factors, including cigarette smoking, alcohol and betel nut consumption and exposure to high-risk human papillomavirus. The risk of HNSCC increases with age, peaking in the seventh and eighth decade, but this varies by anatomical and histological subtype. While several advancements have been made in the treatment of head and neck cancer (HNC) in recent decades, undertaking curative treatment still subjects the majority of HNSCC patients to substantial treatment-related toxicity requiring patients to tolerate a gamut of physical, psychological, and emotional demands on their reserves. In conjunction with other patient-related factors, clinicians involved in treating patients with HNSCC may incorporate advancing chronological age into their decision-making process when determining treatment recommendations. While advancing chronological age may be associated with increased concerns regarding physical treatment tolerability, clinicians may also be concerned about heightened vulnerability in various health and wellbeing outcomes. The available literature, however, does not provide evidence of this vulnerability in patients with advancing age, and, in many instances, older patients self-report greater resilience compared to their younger counterparts. While this data is reassuring it is limited by selection bias and heterogeneity in trial and study design and the absence of a consistent definition of the elderly patient with HNSCC. This narrative review article also includes a review of the measures used to assess HRQL, psychosocial outcomes and unmet needs in elderly or older patients with HNSCC.

## Introduction

Worldwide, head and neck cancer (HNC) accounts for more than 650,000 cases and 330,000 deaths annually and the majority of these are squamous cell carcinomas (HNSCC) (1). Treatment recommendations depend on many factors, including tumor subsite, stage, and pathologic findings, but typically involves surgery and radiation therapy (with or without systemic therapy) either alone or in combination (2). Proceeding along a curative course of treatment for any HNC exposes survivors to the possibility of permanent impairment, including those required for basic human functioning such as chewing, swallowing, tasting and communicating, and loss in these and other domains may also impose significant psychological and emotional challenges (3). It is therefore incumbent upon the HNC physician to weigh the benefits and risks of all therapeutic options, balancing the chance of cure and cost of suboptimal locoregional treatment against the potential toxicity and negative health and wellbeing impacts of curative-intent treatment. Numerous factors weigh into this decision, including performance status, perceived frailty, social supports, individual preferences for pursuing curative treatment and the individual’s physical and mental reserve, among others. Age, whether chronological or the individuals’ perceived biological age, is but one of a host of factors that clinicians may consider during this process.

There is no readily accepted or reliable threshold for defining an “older” HNC patient to incorporate into clinical decision-making, an observation reflecting the degree of variation in ability, functioning and capacity in older adults. As a consequence, tailored treatment recommendations for older patients have been lacking due to an underrepresentation in clinical trials (4). Defining the optimal treatment strategies for these patients, if they differ at all, should be considered a priority for our specialty given the advancing age of the average HNC patient. In the United States for example, the proportion of HNC patients over the age of 65 years is expected to increase from 54% in 2010 to 66% in 2030 (5). In addition, while much attention has been placed on an epidemic of younger patients presenting with human papillomavirus-associated oropharyngeal cancer (HPV+ OPC) the projected increased number of HPV+ OPC cases in the United States from 2016 to 2029 will be driven almost exclusively by patients over 65 years of age (6, 7). Aggressive treatment is definitely feasible for selected older patients with HNC, but reliance on chronological age in isolation may result in over- or under-treatment, and while functional measures may provide superior discrimination in predicting survival and the tolerability of therapy (8–14), they are time intensive and require considerable multidisciplinary expertise (15).

But what other factors do we need to consider for the older HNC patient? And where is the patient’s voice within this complex decision-making process? Do preferences for pursuing radical treatment and cure change with age? Do priorities and preferences for different oncological and functional outcomes change with increasing age? Do older patients demonstrate increased vulnerability to adverse health and wellbeing outcomes when aggressive treatment strategies are employed? Do older HNC patients have more unmet needs following completion of treatment? This narrative review will draw on published research incorporating patient-reported outcomes to examine these questions. Where possible, we have narrowed the scope to focus on studies reporting outcomes in HNSCC; however, many of the studies were very broad in their selection criteria (tumour subsite and histology) and treatment received including any treatment modifications that may have been made to improve treatment tolerance in older patients. Out of necessity, where suitable data was otherwise lacking, some mixed studies have been included in this review. It should also be acknowledged that specific details about patients’ baseline functional performance were lacking, and the presented studies largely included older patients considered fit for a radical course of treatment, introducing a degree of selection bias. Hence, these results cannot be extrapolated to poorly performing older patients.

## Defining “Elderly” Head and Neck Cancer Patients

There is no strict chronological boundary for defining “elderly” patients with cancer. The United Nations and World Health Organization have defined elderly patients as those above 60 (16), while in many developed countries, the designation is tied to retirement, which varies between jurisdictions from 55 to 70 years of age (17). Further refinement of old, including young old (65–74), older old (75–84) and oldest old (≥85) have been proposed and adopted by the National Institute on Aging (18). The European Organization for Research and Treatment of Cancer (EORTC) recommended a cut-off of 70 years of age for their elderly HRQL module, the EORTC QLQ-ELD14 (19). Specifically in HNC, 70 years of age would be considered the most common clinically utilized threshold for decision-making available (4) based on the reported reduction in efficacy of concurrent chemotherapy observed in patients above this threshold (20). However, older patients have typically been underrepresented in HNC and HNSCC clinical trials (4), and caution should be observed in drawing too firm a conclusion given the significant improvement yielded in younger cohorts and the catastrophic complications of treatment failure. While the additional benefit of chemotherapy to locoregional treatment in the elderly remains an unresolved issue, it remains a fertile area of research, one where future studies, in particular, secondary analyses from phase II and III studies, could address questions of efficacy while also providing prospective and high quality data on patient-reported toxicity, HRQL and distress. Such data may provide clinically useful information to aid shared-decision making in older patients with HNSCC.

While there is conjecture and variation surrounding an exact age-related definition of elderly, it is somewhat arbitrary; instead, clarifying core variables such as biological age, medical co-morbidity and frailty lie at the heart of what the clinician is trying to ascertain – whether the patient has sufficient reserve to tolerate the physiological insults of radical treatment. While there has been some tantalizing preliminary work focusing on methods to determine an individuals’ epigenetic clock in HNC patients, it is currently difficult, if not impossible, to measure biological age reliably. This will be discussed in more detail in a subsequent section (21, 22). We acknowledge the limitations of chronological age as a predictor for treatment tolerance, particularly when used in isolation; however, it is a parameter that is commonly used both in clinical and research settings and the purpose of this review was to address variations in health and wellbeing outcomes based on chronological age, rather than other variables, which may provide useful additional data.

## Treatment and Outcome Preferences in Older Patients With HNC and HNSCC

Do older patients with HNC prioritize oncologic outcomes, such as cure, survival and functional outcomes like preserving swallow and communicating, differently than younger patients with HNC? The Chicago Priority Scale is one of the most frequently used instruments in the HNC cancer literature to characterize treatment outcome priorities (23, 24). Patients (or controls) are asked to rank their disease and functional outcome goals in order from first to last for 12 different outcomes; these are listed in **Table 1**. In the first of these studies, which included newly diagnosed HNC patients (n=131) from nine institutions, older patients less frequently prioritized oncological outcomes, such as cure and survival (“living as long as possible) in the top three items. The median age in this study was 59 years (range 29-87 years). In those patients aged <55 years, 55-64 years and >65 years, the frequency of ranking the cure item in the highest three priorities was 98%, 96% and 84%, respectively. While this difference was statistically significant (*p*<0.05), it is clear that the vast majority of older patients place a high value on being cured of their cancer. Interestingly, the older patients did less frequently prioritize length of life or survival, with the “living as long as possible” outcome showing a more notable drop off in the top three ranked items across the same three age groups (73%, 48% and 43%, respectively, *p*=0.01).

**Table 1:** Chicago priorities scale.

Oncological Outcome
“being cured of my cancer”
“living as long as possible”
**Treatment-related outcome**
“keeping my natural voice”
“being able to chew normally”
“being able to swallow all foods and liquids”
“having no pain”
“keeping my appearance unchanged”
“returning to my regular activities as soon as possible”
“having a normal amount of energy for me”
“keeping my normal sense of taste and smell”
“being understood easily”
“having a comfortably moist mouth”

List et al. subsequently reported results for a larger sample of recently diagnosed, but untreated HNC patients (n=247) in parallel to a control sample (n=131) of non-cancer patients with no personal experience of HNC in close friends or relatives. Similar findings were observed, with most of the “older” group of patients in this series (older was defined by the median split at >58 years) placing the cure outcome in the top three outcomes (≤58: 93% vs >58: 87%). However, older patients also prioritized the “keeping appearances unchanged” item in the top three items more frequently than younger patients (13% vs 5%). Similar observations for both cure, length of life and appearances were observed in the control group.

The Chicago Priority Scale was also used to assess preferences in a more contemporary cohort of patients from John Hopkins, which importantly included many patients with HPV+ OPC (88/150, 59%) (25). Investigators conducted a one-time interview of patients with a mix of HNSCC tumor sites and treatments (surgery ± adjuvant therapy 70%, primary CRT/RT 30%), and the time since treatment completion also varied (median 7 months, IQR 1.5-23.5 months). The median age in this study was lower than in both prior studies (median 54, IQR 54-67, range 26-90). Cure was considered the highest priority at all ages with no differences reported with increasing decade of life. Like both studies by List and colleagues, survival (“living as long as possible”) was less important with increasing age, a finding unchanged after adjustment for HPV status, disease stage and HRQL scores. In the full cohort, the survival outcome was ranked the second most important outcome after cure (median rank 2.5, IQR 2-9), in those aged 75 or over the median rank of the survival outcome was only 6 (IQR 2-11). Nonetheless, there was wider variability in the ranking of the survival item in older patients. In this study, treatment regret using the Ottawa Decision Regret Scale also did not vary by age. In a smaller series of 37 HPV+ OPC patients also from John Hopkins which included patients aged <60 years (23/37,62%), 60-69 years (7/37, 19%) and ≥70 years (7/37, 19%), Windon et al. noted few changes in treatment priorities from baseline (median 1 month after diagnosis, IQR 1-2 months) to post-treatment (median 8 months after treatment, IQR 7-10 months) with the exception that the chewing normally item was increasingly prioritized with older age in the post-treatment setting compared to its initial ranking.

Patient preferences have been appraised in other settings, for instance, where survival may be traded for reduced toxicity or organ preservation. In a Canadian study, Brotherston et al. reported that 51 previously-treated (≥3 months earlier) oropharyngeal cancer patients were willing to accept very little reduction in survival for a reduction in toxicity (which, in this case, was omitting chemotherapy), and when analyzed by age (median age 58 years; 60-69 years: 19/51, 37%, ≥70 years 4/51, 8%) there were no differences in cure sacrifice thresholds (26). In patients treated for advanced laryngeal cancers, at least one study has shown that age was not significantly associated with variations in the willingness to trade laryngeal preservation for survival (27).

In the studies reviewed, cure remains a high priority for HNC patients irrespective of age. Treatment outcome priorities and preferences studies provide some useful broad concepts to guide clinicians but are not a surrogate for eliciting preferences from patients at an individual level, which must remain first and foremost in any shared decision-making model.

## Health-Related Quality of Life

A large number of studies have examined age-related variations in health-related quality of life (HRQL) outcomes; however, these reports vary in design (longitudinal or cross-sectional) and how age has been included as a variable (categorical or continuous) in the analysis. Relevant studies have been tabulated for ease of reference, including longitudinal (**Table 2**) and cross-sectional studies (**Table 3**) that have modeled age as a categorical variable, single-arm studies enrolling only elderly patients (**Table 4**) and those that have modeled age as a continuous variable (**Table 5**). Yet another strategy in the literature has been to compare the outcomes of elderly patients to age-matched population controls, allowing the authors to account for changes expected with normal aging.

**Table 2:** Prospective studies reporting health-reported quality of life in elderly patients^a^.

Author	Year	n	Location	Handling of age^a^	H&N subsite	Treatment	Instruments	Study design	Global QoL findings	Other HRQL findings
***Global QoL favoring older***										
Berg et al. (60)	2021	311	Sweden	<70 vs ≥70 years; <80 vs ≥80 years	OCC, 31% OPC, 35% LC, 17% Other, 27%	Sx, 15% Sx + aRT/CRT, 22% RT, 19% RT/CRT, 44%	QLQ-C30 QLQ-H&N35 QLQ-ELD14	Prospective: pretreatment, 3m, 6m, 12m post Rx	Global QoL favored elderly at 3 months only: ≥70 (<70 vs ≥70: 52 vs 61, p=0.006); and ≥80 (<80 vs ≥80: 54 vs 67, *p*=0.024)	Most HRQL scores similar or better in older patients, with exception of PF; older patients less appetite loss and FD; oldest (≥80 years) worse fatigue, RF and feeling ill at 12months
Citak and Tulek (61)	2013	54	Turkey	<60 vs ≥60 years	LC, 67% OCC, 19% Pharynx, 15%	All received RT Sx, 69% Any RT, 63% Any CRT, 38%	QLQ-C30 QLQ-H&N35	Prospective: pretreatment, end of RT, 1m and 3m post RT; factors analysed at end of RT;	Global QoL better in older (53 vs 41, *p*=0.021)	Only senses problems (25 v 47, *p*=0.011) and weight loss (59 v 87, *p*=0.017) SS and worse in younger patients; remainder not SS
***Global QoL favoring younger***										
Aoki et al. (62)	2019	172	Japan	<75 vs ≥75 years	OCC, 100%	Sx, 100% aRT/CRT, 10%	FACT-H&N	Prospective: pretreatment, 1, 3 and 6 months post Rx	Global score similar at start but younger better at 6 months posttreatment in favour of younger (106 vs 97)	SWB and HNC additional concerns worse at 6m in elderly group; non-elderly group showed improvement at 6m in PWB, EWB and FWB, while the elderly group did not
***Age associated with other HRQL domain scores other than global QoL***										
De Graeff et al. (63)	2000	107	The Netherlands	<60 vs ≥60 years	OCC, 46% OPC, 6% HPC, 3% LC, 43% Other, 2%	Sx alone, 27% RT, 45% Sx + aRT, 28%	QLQ-C30 QLQ-H&N35	Prospective: pretreatment, 6, 12, 24, 36m post Rx	Global QoL not SS (absolute values not reported)	PF worse in older SS (absolute difference not reported); remaining items/scales not SS
De Graeff et al. (64)	2000	153	The Netherlands	<60 vs ≥60 years	4 groups based on site and treatment	LC, RT, 44% OCC/OPC, Sx, 22% OCC/OPC, Sx + aRT, 26% HPC/LC, Sx + aRT - 7%	QLQ-C30 QLQ-H&N35	Prospective: pretreatment, 6m and 12m post Rx;	Global QoL not SS	Older patients’ worse fatigue, PF, social eating and speech; remainder NS
Derks et al. (65)	2004	183	The Netherlands	45-60 vs ≥75 years	OCC, 48% Pharynx, 37% LC, 15%	Sx, 23% Sx ± adj RT, 48% RT, 16% CRT, 11%	QLQ-C30 QLQ-H&N35	Prospective: pretreatment, and 3m, 6m and 12m post Rx	Global QoL similar at all time points;	PF better* in younger at baseline (78 v 69), 3m (65 v 57), 6m (72 vs 62), not SS at 12m (70 vs 62); Pain worse in younger (37 vs 17) only at 6m post; remainder NS or trivial differences
Dziegielewski et al. (66)	2013	81	USA	<55 vs ≥55 years	OPC	All TORS aRT/CRT, 87%	HNCI	Prospective: pretreatment, and 3w, 3m, 6m and 12m post Sx	Global QoL NS (81 v 70, *p*=0.11)	No difference in other functional outcomes; younger patients reported lower attitude (satisfaction) on speech (71 vs 88) and aesthetic (73 v 91)
Funk et al. (67)	2012	337	USA	≤58 vs ≥59 years		Mixed subsite; Mixed Rx	SF-36 HNCI	Prospective, multiple time points; baseline at pretreatment; current study reports at 5 years;	Age not SS (72.0 vs 76.0, NS)	Older age SS better aesthetics, social disruption, mental health and depressive symptoms but worse physical health
Hammerlid et al. (68) and Bjordal et al. (69)	2001	357	Norway Sweden	<65 vs ≥65 years	OCC, 38% LC, 24% OPC, 10% HPC, 8% Other, 20%	–	QLQ-C30 QLQ-H&N35	Prospective: pretreatment to 12m	Global QoL not SS at baseline (68 v 70) or change from baseline to 12m (3 v 1)	*At baseline:* Older tended to report worse scores, but only significant for PF, constipation, dyspnoea and coughing; Older patients better EF *12m post:* Older higher sexuality and sticky saliva problems; Older better RF, EF, but reported *Change from baseline to 12m* Older better RF and smaller increase in dry mouth scale; Older larger changes in senses (16 v 10), sexuality (17 v 4) and nutritional supplements (10 v 5)
Hammerlid et al. (68)	2001	232	Sweden	<65 vs ≥65 years	OCC, 32% Pharynx, 28% LC, 29% Other, 22%	Sx alone, 5% Sx + aRT, 25% Sx + aCRT, 9% CRT, 31% RT, 30%	QLQ-C30 QLQ-H&N35	Prospective: pretreatment, 6 times in year 1, then year 3	Global QoL not SS (76 vs 79)	Older reported worse mucous production, more problems with sexuality and feeling ill; but less financial difficulties and better EF and less insomnia; SS in PF favoring younger (89 vs 81)
Reeve et al. (70)	2016	587	USA	<50 vs 50-64 vs 65-74 vs ≥75 years	OCC, 53% LC, 38% Pharynx, 10%	Sx, 57% RT, 77% Chemo, 41%	FACT-H&N	Prospective: (baseline = mean 3m post diagnosis, + 2 other time points	Total FACT-H&N not reported	Older patients reported better PWB, EWB, FWB and fewer symptoms than younger patients but not SWB
Rettig et al. (71)	2016	1653	USA	<67 vs 68-72 vs 73-77 vs >78 years	LC, 38% OCC, 23% OPC, 18% Lip 12% HPC, 4% Other, 5%	RT, 59%	Combined SF-36 PCS/MCS score or Veterans RAND	Prospective, baseline within 5 years of diagnosis and follow up with 10y posttreatment	–	*Composite PCS/MCS estimated differences:* UVA (*p*<0.001); Ref group <67 68-72: 3.9 (1.5,6.3) 73-77: 2.6 (-.1,5.1) >78: -2.0 (-4.6,0.5) MVA: (*p*=0.01) Ref group <67 68-72: 1.9 (-0.1,3.9) 73-77: 1.0 (-1.0,3.0) >78: -1.3(-3.4,0.9)
Van Der Schroeff et al. (72)	2006	59	The Netherlands	45-60 vs ≥70 years	OCC,51% Pharynx, 30% LC, 19%	Mixed site; mixed Rx	QLQ-C30 QLQ-H&N35	Prospective: pretreatment, 12m, 3-6y	Global QoL NS at all time points	Older group worse PF at 12m (81 v 66) and 3-6y (81 vs 67); older group worse symptoms on QLQ-H&N35, including social eating at 12m, swallowing at 12m and 3-6y and speech 12m and 3-6y
***Age not associated with any HRQL domain scores***										
Bozec et al. (73)	2008	65	France	<70 vs ≥70 years	Mixed subsite (61/65 SCC) OCC, 87% HPC, 11% PNS, 2%	All Sx with microvascular reconstruction; Preop RT 20% aRT 65%	QLQ-C30 QLQ-H&N35	Prospective: pretreatment, 6m, 12m; analysis of factors limited to 6m post	Global QoL at 6m similar (63 vs 67; *p*=0.74)	No differences in functioning scales or other domains
Bozec et al. (74)	2018	60	France	<70 vs ≥70 years	Primary or recurrent OPC SCC	All Sx and RFFF aRT, 41% aCRT, 35%	QLQ-C30 QLQ-H&N35 (included other PROMs)	Prospective: pretreatment and ≥12m post Rx; analysis of factors after treatment	Global QoL not SS (absolute values not reported; *p*=0.43)	No differences in functioning scales or other domains
Bozec et al. (75)	2019	200	France	<65 v >65 years	OCC, 41% OPC, 20% HPC, 10% LC, 28%	All Sx; aRT, 21% aCRT, 24%	QLQ-C30 QLQ-H&N35	Prospective: pretreatment and 6m post Rx; analysis of factors after treatment	Global QoL not SS (absolute values not reported; *p*=0.89)	No differences in functioning scales or other domains
Derks et al. (76)	2003	129	The Netherlands	45-60 vs ≥75 years	OCC, 64% OPC/HPC, 27% LC, 9%	All Sx aRT, 69%	QLQ-C30 QLQ-H&N35	Prospective: pretreatment and then 2-3 months, adj RT completed at beginning of RT	Global QoL not SS	No other domains different either at baseline or in changes from baseline
Durmus et al. (77)	2014	22	USA	<55 vs >55 years	CUP	All TORS + aRT/CRT	HNCI	Prospective: pretreatment, 3w, 3m, 6m, 12m post Sx	Age NS for global QoL	Age NS for all items/scales
Segna et al. (78)	2018	30	Italy	<70 vs ≥70 years	OCC	All reconstructive microsurgery aRT, 43% aCRT 17%	SF-36/SF-12	Prospective: pretreatment and 12m post Sx	–	No differences in any domains or composite scores
Yin et al. (79)	2020	294	China	<60 vs ≥60 years	HPV OP SCC	Sx alone, 35% Sx + aRT, 20% RT, 45%	QLQ-C30 QLQ-H&N35	Prospective: pretreatment and 3-6m post Rx; factors analyzed post Rx	Age not a factor on MVA for global QoL	Age not a factor for other reported outcomes
Yoshimura et al. (80)	2009	56	Japan	≤65 vs >65 years	OCC	OCC treated with LDR-BT	QLQ-C30 QLQ-H&N35	Prospective: pretreatment, 3m, 6m and 12m post LDR-BT	Age NS for global QoL	Age NS for any outcome

aRT/CRT, adjuvant radiotherapy/chemoradiotherapy; CRT, chemoradiotherapy; EF, emotional functioning; EWB, emotional wellbeing FACT-HN, Functional Assessment of Cancer Therapy-Head &Neck; FWB, functional wellbeing; H&N, head and neck; HNC, head neck cancer; HNCI, Head Neck Cancer Inventory; HRQL, health-related quality of life; HPC, hypopharyngeal cancer; LC, laryngeal cancer; MCS, Mental Component Summary; MVA, multivariate/variable analysis; NS, not statistically significant; OC, oral cavity; OPC, oropharyngeal cancer; PCS, Physical Component Summary; PF, physical functioning; PWB, physical wellbeing; QLQ-C30, European Organization for Research and Treatment of Cancer Quality of Life Questionnaire-Core 30; QLQ-ELD14, European Organization for Research and Treatment of Cancer Quality of Life Questionnaire-Elderly Cancer Patients module; QLQ-H&N35, European Organization for Research and Treatment of Cancer Quality of Life Questionnaire-Head and Neck module; QOL, quality of life; RF, Role functioning; RT, radiotherapy; Rx, treatment; SF-12, Short Form-12 Health Survey; SF-36, Short Form-36 health survey; SS, statistically significant; SWB, social wellbeing; Sx, surgery; UVA, univariate/variable analysis.

a Definition of elderly and age groups based on categorizations used in the analysis of Global QoL scores.

**Table 3:** Cross-sectional studies reporting health-reported quality of life in elderly HNSCC cohorts. .

Author	Year	n	Location	Handling of age	H&N subsite	Treatment	Instrument	Study design	Global QoL findings	Other findings
Alicikus et al. (81)	2008	110	Turkey	≤60 vs >60 years	LC, 58% NPC, 18% OC/OPC, 14% Other, 6%	All received RT/CRT RT, 42% CRT, 13% aRT, 43% aCRT, 2%	QLQ-C30 QLQ-H&N35	Cross-sectional (median 29m, range 4-155m)	Global QoL not SS (69 v 70)	PF, RF, SF, CF not different (EF not reported); younger patients reported more problems with teeth (44 vs 18) and opening mouth (30 v 15);
Baxi et al. (82)	2018	185	USA	<65 vs ≥65 years	All HPV+ OPC	All received RT/CRT definitive, 86% Adjuvant, 14%	EQ-5D, QLQ-H&N35	Cross-sectional (>12 months from RT)	Global QoL (EQ-5D VAS) similar (86 vs 81; *p*=0.20)	Older patients worse mobility (EQ-5D), remainder of EQ-5D similar; worse social eating (ED 11.1, *p<*0.0001) and coughing (ED 11.7, *p*=0.009)
Bonzanini et al. (83)	2020	90	Brazil	<62 vs ≥62 years	OCC/OPC, 60% LC, 34% HPC, 6%	All received RT Sx + aRT, 19% Sx + aCRT, 40% RT, 7% CRT, 34%	UW-QoL	Cross-sectional	Mean scores 60 vs 74; age SS on MVA	Younger patients reported worse pain (64 v 81), appearance (70 vs 81), swallowing (56 v 75), chewing (55 v 70), shoulder problems (56 v 84), saliva (43 v 59)
Bozec et al. (84)	2020	64	France	<80 vs >80 years (inc ≥70 years)	All OC/OPC	All free flap reconstruction Sx alone, 22% aRT, 61% aCRT, 17%	QLQ-C30 QLQ-H&N35 QLQ-ELD14	Cross-sectional (>12 months after Sx)	Global QoL not SS (value NR)	Mobility score on QLQ-ELD14 favouring younger 16.7 vs 22.0, *p*=0.004); remainder not SS
Dwivedi et al. (85)	2012	55	United Kingdom	<60 vs ≥60 years	OCC and OPC	All Primary Sx Sx alone, 11% Sx + aRT, 49% Sx + aCRT, 40%	UW-QoL	Cross-sectional	–	Mean composite score of 12 domains used - younger worse (70 vs 80, p=0.01);
Infante-Cossio et al. (86)	2009	128	Spain	<65 vs >65 years	OCC, 55% OPC, 45%	NR	QLQ-C30 QLQ-H&N35	Collected at time of diagnosis	Age not SS for global QoL (median 83.3 vs 83.3, *p*=NS)	PF, CF, fatigue, pain worse in older patients (all *p*<0.05, based on median scores, all scores <10 except for fatigue)
Laraway (87)	2012	638	United Kingdom	<55 vs 55-64 vs 65-74 vs ≥75 years	All OCC	Sx, 99% Sx + aRT/CRT, 32% CRT/RT, 1%	UW-QoL	Cross-sectional; “closest to 1 year after surgery”	Patients ≥65 reported better overall QoL (proportion reporting good or better, *p*<0.001)	Many domains favored the older age groups in both the physical and socioemotional domains
Morimata et al. (88)	2013	100	Japan	65 vs >65 years	Maxillectomy, 50% Mandibulectomy, 50%	Maxillectomy, 54% aRT Mandibulectomy, 32% aRT	UW-QoL (v4.0)	Cross-sectional	Age not SS for global QoL in either maxillectomy *(p*=0.80) or mandibulectomy population (*p*=0.54)	*Maxillectomy:* younger patients more anxious (*p*=0.01) *Mandibulectomy:* younger patients better swallowing (*p*=0.01), saliva (p=0.04) and mood (*p*=0.03)
Pierre et al. (89)	2014	80	France	<70 vs >70 years	OCC, 46% OPC, 54%	All Surgical patients with microvascular reconstruction aRT, 69% naRT, 11%	QLQ-C30 QLQ-H&N35	Cross-sectional	Age NS for global QoL	Age NS for all items/scales
Pourel et al. (90)	2002	113	France	≤62 vs >62 years	All OPC	Sx + RT, 23% BT + RT, 43% RT, 33%	QLQ-C30 QLQ-H&N35	Cross-sectional (≥2y post RT)	Global QoL not SS (66 vs 64, *p*=0.70)	Other functional scales, fatigue did not differ; pain worse in younger (32 vs 21, *p*=0.03)
Silvieri et al. (91)	2011	289	Portugal	40-60 vs ≥65 years	*Younger:* LC, 31% OC, 16% OPC, 11% *Older:* LC, 30% OC, 13% OPC, 3%	*Younger:* Sx, 71% RT, 8% CT, 10% CRT, 11% *Older:* Sx, 79% RT, 14% CT, 2% CRT, 5%	QLQ-C30 QLQ-H&N35	Cross-sectional (3-9m post Rx)	Global QoL similar across all groups when analyzed by gender	Some small-sized differences reported (i.e. younger males less constipation, worse financial difficulties; older females worse PF (medium-sized), remainder differences small-sized Few significant differences in QLQ-H&N35 in young vs older males, except for higher sexuality problems in older males (26 vs 39); for females, only speech problems were clinically significant and borderline SS in younger (35 v 18)
Verma et al. (92)	2019	58	USA	<65 vs ≥65 years	HNSCC, further details NR	Definitive RT/CRT, 50% Sx + aRT/naRT, 50% CT, 72%	QLQ-C30, Dental health, shoulder function	Cross-sectional	Global QoL NS	Older patients’ better RF (95 vs 76), EF (89 vs 79) and lower pain (4 vs 29), insomnia (8 vs 36) an financial problems (8 vs 38)
Wells et al. (93)	2015	289	United Kingdom	<45 vs 45-54 vs 55-64 vs 65-74 vs ≥75 years	OCC, 34% OPC, 20% LC, 33% Other, 13%	Sx alone, 26% Sx + aRT, 11% Sx + CT, 1% Sx + aCRT, 23% RT, 17% CRT, 18%	QLACS	Cross-sectional (≥3m but <5y post Rx)	*Generic QoL:* younger not SS (83.8 vs 82.9 vs 79.8 vs 70.0 vs 73.3, *p*=0.118); higher score = worse) *Cancer-specific QoL:* younger worse (43.8 vs 46.8 vs 40.5 vs 34.1 vs 31.5, *p*<0.001) (higher score = worse)	–
Williamson et al. (94)	2011	41	USA	<70 vs ≥ 70 years	All LC	Sx + aRT/CRT, 27% RT, 63% CRT, 10%	UW-QoL	Cross-sectional	Age NS for overall QoL (even when ≥75 was compared)	Age NS for other items/scales
Woodard et al. (95)	2007	33	USA	≤65 vs >65 years	All LC/HPC	Laryngectomy, 100% ± adj and previous RT/CRT	HNCI	Cross-sectional (mean 37m)	Older SS better global QoL 75 vs 54)	Older SS better in all 4 domains (speech, eating, social disruption, aesthetic)

aRT/CRT, adjuvant radiotherapy/chemoradiotherapy; CF, cognitive functioning CRT, chemoradiotherapy; ED, estimated difference; EF, emotional functioning; EQ-5D, EuroQoL 5-Dimension; H&N, head and neck; HNCI, Head Neck Cancer Inventory; HPC, hypopharyngeal cancer; LC, laryngeal cancer; MVA, multi- variate/variable analysis; NR, not recorded; OC, oral cavity; OPC, oropharyngeal cancer; QLACS, Quality of Life of Adult Cancer Survivors; QLQ-C30, European Organization for Research and Treatment of Cancer Quality of Life Questionnaire-Core 30; QLQ-ELD14, European Organization for Research and Treatment of Cancer Quality of Life Questionnaire-Elderly Cancer Patients module; QLQ-H&N35, European Organization for Research and Treatment of Cancer Quality of Life-Head and Neck module; QoL, Quality of life; PF, physical functioning; RF, Role functioning; RT, radiotherapy; SF, Social functioning; SS, statistically significant; Sx, surgery; UW-QoL, University of Washington Quality of Life instrument.

**Table 4:** Studies reporting health-related quality of life outcomes enrolling only elderly patients with HNSCC.

Author	Year	n	Location	Ages included	Participants	Instrument	Study design	Findings
Dimovska et al. (28)	2016	34	United Kingdom	≥80 years	Sx with microsurgical reconstruction	UW-QoL (v4)	Cross-sectional	Overall score represented mean of the physical and socioemotional domains, 78.7; physical function mean score, 76.52; socioemotional mean score, 80.9
Fang et al. (29)	2014	59	China	≥70 years	Comparison of surgical patients with and without free flap reconstruction	UW-QoL (v4.0)	Cross-sectional (≥12m post Rx)	Mean composite QoL similar (77.5 vs 72.1, *p*=0.231); SS differences favoring free flap group in chewing (69.0 vs 57.0)
Ferri et al. (10)	2019	39	Italy	>75 years	Sx with microsurgical reconstruction	SF-36	Cross-sectional	Average score of all scales was 68.3; MCS, 44.6; PCS, 46.5 Highest scale: Physical, 76.92; Lowest scale: energy/fatigue, 60.25 Compared to general population: PCS was higher in this group (46.53 vs 37.85)
Ruhle et al. (30)	2021	50	Germany	≥65 years	Definitive and adjuvant RT/CRT	QLQ-C30 QLQ-H&N35	Cross-sectional (≥12m post Rx)	Median global QoL (66.7) comparable to German age- and gender matched cohort (65.3); Global QoL similar between definitive and adjuvant CRT (median 75 vs 66.7, *p*=0.219); HPV+ had superior global QoL to HPV-

HPV, human papillomavirus, MCS, Medical Component Summary; PCS, Physical Component Summary; QoL, quality of life; QLQ-C30, European Organization for Research and Treatment of Cancer Quality of Life Questionnaire-Core 30; QLQ-H&N35, European Organization for Research and Treatment of Cancer Quality of Life Questionnaire-Head and Neck module; Rx, treatment; SF-36, Short Fom-36 Health Survey; Sx, surgery; UW-QoL, University of Washington Quality of Life instrument.

**Table 5:** Studies reporting age-related differences in health-related quality age in HNSCC (age analyzed as a continuous variable).

Author	Year	n	Location	Age	H&N Subsite	Treatment	Instrument	Study Design	Global QoL findings	Other findings
***Prospective***										
Borggreven et al. (31)	2007	80	The Netherlands	Mean 58 years (23-74 years)	OCC, 47% OPC, 53%	All composite resections + microvascular reconstruction aRT/CRT, 93%	QLQ-C30 QLQ-H&N35	Baseline and 6m and 12m post Rx	Older age associated with worse global QoL at 6m (*p*=0.041), but not at 12m (raw scores not provided)	NR
Hu et al. (32)	2020	105	China	Mean 60.3 years (NR)	LC, 69% HPC, 7% NPC, 6% OCC, 6% OPC, 3% Other, 9.5%	All Sx	QLQ-C30	Prospective; baseline, 3-9 days post and 1m post Sx	Older age SS worse global QoL at 1m post only	Older age associated with worse PF at baseline (B=-0.089) and 1m (B=-0.047)
Ronis et al. (33)	2008	316	USA	Mean 58.6 years (28-86 years)	OC, 22% Pharynx, 54% LC, 25%	Any Sx, 51% Any RT, 86% Any CT 65%	SF-36; HNQoL	Prospective; baseline and 12m;	–	Age negatively and SS with PCS (SF-36); improved emotion domain HNQoL; NS for all other domains
Singer et al. (34)	2014	133	Germany	Mean 58 years (38-88 years)	All LC	All laryngectomy aRT, 13% No aRT, 61% Unknown, 26%	QLQ-C30 QLQ-H&N35	Prospective, baseline, discharge, end of rehab, 12m post; factors analyze as difference at 12m vs baseline; age as continuous variable;	Age NS for global QoL;	Age not associated with any outcome
***Cross-sectional***										
Allison et al. (35)	1998	188	Canada	Mean 64.6 years (range 34-91 years)	OCC, 40% Pharyngeal, 30% LC, 30%	Sx alone, 26% RT alone 33% Sx + aRT, 42%	QLQ-C30	Cross-sectional (mean time since treatment 22m, range 1-168m)	Global QoL not correlated on UVA model (*r*= -0.03, *p*=0.65); but older age significantly worse on MVA model using clinical and sociodemographic variables (parameter estimate -0.04, SE 0.01, *p*=0.0003)	
McDowell et al. (36)	TBD	136	Australia	Mean 61 years (42-87 years)	All HPV OPC	RT alone, 12% CRT, 88%	QLQ-C30	Cross-sectional (≥12m post Rx); mean 3.0y from treatment;	Age NS for global QoL (*p*=0.579)	Other domains not analyzed by age; age did not differ in a low and high functioning subgroup based on a cluster analysis of QLQ-C30 functioning domains
Mehanna and Morton (37)	2006	43	New Zealand	Mean 64 years (NR)	OCC, 14% LC, 52% Pharyngeal, 17% Other, 17%	Mixed site Mixed Rx	Auckland QoL Questionnaire	Cross-sectional	Age NS for Global QoL (aggregated life satisfaction score)	–
Raemakers et al. (38)	2011	396	The Netherlands	Mean 63.2 years (20-99 years)	OCC, 13% Pharyngeal, 29% LC, 32% Other 25%	All patients RT Sx, 35% CT, 14%	EQ-5D	Cross-sectional (median 20m post Rx)	Age not a factor on MVA model for either utility or VAS score	–
Rogers et al. (39)	2009	65	USA	Mean 60years (NR)	OCC, 25% Pharyngeal, 40% LC, 20% Other, 15%	All Sx RT, 82% CT, 40%	FACT-H&N	Cross-sectional (>6m post Rx)	Age not SS factor for FACT-G or FACT-H&N	Older patients better EF

H&N, head and neck; QOL, quality of life; LC, laryngeal cancer; HPC, hypopharyngeal cancer; NPC, nasopharyngeal cancer; OC, oral cavity; OPC, oropharyngeal cancer; Sx, surgery; QLQ-C30, European Organization for Research and Treatment of Cancer Quality of Life Questionnaire-Core 30; ART, adjuvant radiotherapy; CRT, chemoradiotherapy; QLQ-H&N35, European Organization for Research and Treatment of Cancer Quality of Life Questionnaire-Head and Neck module; Rx, treatment; NR, not recorder; RT, radiotherapy; CT, chemotherapy; SF-36, Short Form-36 Health Survey; HNQOL, Head Neck Quality of Life instrument; PCS, Physical Component Summary; UVA, univariate/variable analysis; NS, not statistically significant; MVA, multivariate/variable analysis; EQ-5D, EuroQoL 5-Dimension instrument; VAS, visual analog scale; FACT-HN, Functional Assessment of Cancer Therapy-Head and Neck module.

For this narrative report, we have focused and drawn attention to studies which have measured HRQL outcomes longitudinally with pretreatment assessments as their baseline, as of the numerous available studies, they are best placed to assess and report treatment-related differences across the age continuum. We have also drawn specific attention to those studies demonstrating significant age-based differences in HRQL, whether these differences have favored either the older or younger patients. However, it should be noted that these studies have also varied in study design from small underpowered studies to studies with very large numbers of patients while also using a variety of approaches to model age, including different age-based thresholds, hampering direct comparison. There is also variation in the instruments used and their assessment times. While the European Organization for Research and Treatment of Cancer (EORTC) modules, discussed below, have been the most frequently used measures in these studies, other instruments have been used, and cross-study comparisons are difficult given differences in their content, response options and the time frame with which individual instruments ask patients to consider how they have performed for a given item (i.e., “during the last week” or “during the past 4 weeks”). These are important differences to consider when comparing studies.

### Health-Related Quality of Life Instruments

A variety of instruments are available to assess HRQL in HNC populations (**Table 6**). These include: (1) generic measures, which may also be used in non-cancer populations, such as the Short Form Health Surveys (SF-36, SF-12) (41, 42) or the EuroQoL modules (40); (2) generic cancer measures, such as the EORTC quality of life core questionnaire (QLQ-C30) (43) and the FACT-General (FACT-G) instrument (45); (3) head and neck cancer-specific instruments including the University of Washington Quality of Life Questionnaire (UW-QOL) (53), the EORTC Quality of Life Questionnaire Head and Neck Module (EORTC-QLQ-H&N35/43) (47, 48), and the Functional Assessment of Cancer Therapy Head and Neck (FACT-H&N), which includes the FACT-G and an additional HNC concerns module (49). Many of these HRQL tools are recommended and available for use in elderly HNC patients, but development and validation studies have not always included sufficient numbers of elderly patients (54). Many authors have recognized this problem, and while steps have been taken to develop psychometrically sound tools to measure HRQL in elderly cancer patients, such as the QLQ-ELD14/15 module developed by the EORTC (19, 55), often these tools do not address cancer-specific challenges faced by older HNC patients. For instance, HNC patients were not represented in the development phase of the QLQ-ELD14 (55). Thus, in selecting instruments for any given study investigating HRQL in elderly HNC patients, choices will largely depend on the clinical context and no single instrument is likely to assess all issues relevant to elderly HNC patients.

**Table 6:** Health-related quality of life instruments often used in HNC studies.

Instrument	Global QoL measure	No. of items contributing to global score	Domains	Validation cohort	Target population
***Generic***					
EuroQoL 5-Dimension (EQ-5D) (40)	Yes	1 (VAS)	Mobility, self-care, usual activities, pain/discomfort, anxiety/depression	General population	≥12 years
SF-12 Health Survey (SF-12) (41)	No	–	Physical functioning, role physical, bodily pain, general health, vitality, social functioning, role emotional, mental health^a^	General population	≥18 years
SF-36 Health Survey (SF-36) (42)	No	–	Physical functioning, role physical, bodily pain, general health, vitality, social functioning, role emotional, mental health^a^	General population	≥18 years
***Cancer-Specific***					
EORTC QLQ-C30 (43)	Yes	2	Physical, role, emotional, cognitive, social functioning; fatigue, pain, nausea and vomiting, dyspnea, insomnia, appetite loss, constipation, diarrhoea; financial impact	Version 1: 36-91 years (mean 63 years) (43); Version 3: 22-91 years (mean 63 years) (44)	≥30 years
FACT-G (45)	Yes	27	Physical, social, emotional, functional wellbeing	Item generation 27-86 years	≥18 years
***Head and Neck Cancer-Specific***					
Auckland Quality of Life Questionnaire (AQLQ) (46)	Yes	1	Social, family and physical functioning	Range not reported (mean 62 years)	Not specified
EORTC QLQ-H&N35/43 (47, 48)	No	–	H&N35: pain, swallowing, senses, speech, social eating, social contact, sexuality; and teeth, mouth opening, dry mouth, sticky saliva, coughing, felt ill, pain killers, nutritional supplements, feeding tube, weight loss, weight gain H&N43: pain in the mouth, swallowing, teeth, dry mouth and sticky saliva, senses, speech, body image, social eating, sexuality, problems with shoulder, skin problems, fear of progression; and opening mouth, coughing, social contact, swelling in the neck, weight loss, wound healing, neurological problems	H&N35 phase 3: 32-89 years	18-88 years
FACT-HN (49)	Yes	37	FACT-G domains plus HNC-specific issues	17-82 years (mean 58 years)	≥17 years
Head and Neck Quality of Life Instrument (HNQOL) (50)	Yes	20	Communication, discomfort, eating, and emotion	Age range not reported (77.4% > 50 years)	≥18 years
Head and Neck Cancer Inventory (HNCI) (51)	Yes	1	Speech, eating, social disruption and aesthetics	<55-≥75 years (mean 61.3 years); 15% ≥75 years	Not specified
UW-QoL v4 (52)	Yes	1	Physical and social-emotional function; 12 single item symptom scores: pain, appearance, activity recreation, swallowing, chewing, speech, shoulder, taste, saliva, mood, anxiety	Version 1: 23-83 years (mean 55 years); 5/75 (6.5%) ≥70 years	≥18 years

EORTC QLQ-C30, European Organisation for the Research and Treatment of Cancer Quality of Life Questionnaire-Core 30; FACT-G, Functional Assessment of Cancer Therapy-General; FACT-HN, Functional Assessment of Cancer Therapy-Head & Neck module; HRQL, health-related quality of life; UW-QoL,- University of Washington Quality of Life Questionnaire; VAS, visual analogue scale.

a Domain scores are used to calculate a Physical Component Summary score and a Mental Component Summary score.

### Global Quality of Life

Despite their limitations (56, 57), global measures of HRQL (referred to hereafter as global QoL) provide a straightforward means of assessing the overall impact of cancer and its treatment (56–59). Most prospective studies including measures of global QoL have found no significant differences between younger and older HNC patients (**Table 2**) (63, 64, 66–80) or have found significantly better self-reported global QoL in older patients (60–62). Findings from cross-sectional studies are similar (**Table 3**).

Berg et al. recently investigated longitudinal HRQL trajectories in 311 Swedish HNC patients. The sample included patients with various tumor sites, stages of disease and treatment; 37% had surgery and 85% received RT or CRT, either as definitive (63%) or adjuvant (22%) treatment (60). HRQL measures (EORTC QLQ-C30, QLQ-H&N35, QLQ-ELD14) were completed at diagnosis, then at three, six and 12 months from the commencement of treatment. The authors undertook two sets of analyses to compare younger and older patients. The first compared younger and older patients (<70 vs ≥ 70 years), the second compared younger and oldest patients (<80 vs ≥80 years). Despite similar global QoL at baseline, older patients reported better global QoL (<70 vs ≥70 years: 52 vs 61, *p*=0.006) three months after treatment, a difference of borderline clinical significance (96, 97); the oldest group (<80 vs ≥80 years: 54 vs 67, *p*=0.024) also fared better on global QoL at three months. Differences at three months were no longer evident at subsequent follow-ups. Citak et al. reported a prospective study of 54 Turkish patients, all of whom underwent radiotherapy (adjuvant treatment in 69%) (61). HRQL was captured with the EORTC QLQ-C30 and QLQ-H&N35 at baseline, at the end of RT, and one and three months after treatment. Global QoL was better in the older cohort (<60 vs ≥60 years: 53 vs 41, *p*=0.021) at the end of RT. While clinically relevant (96, 97), score differences at baseline were not described, so these results should be interpreted with caution.

Conversely, in a study reporting HRQL in elderly Japanese patients with oral cancer, Aoki et al. reported lower global QoL in older patients six months after treatment (62). In this study, 172 patients (≥75 years, n=43) completed the FACT-H&N at baseline, at treatment completion and one, three- and six-months post-treatment. While differences were small at the earlier time points, older patients reported worse global QoL scores at six months post-treatment (graphical interpretation, 106 vs 97, *p*=0.009), which falls into the range of a clinically meaningful difference (98). Interestingly, younger patients showed continued improvement from completion of treatment, whereas older patients plateaued at 1-month post-treatment.

### Other Health-Related Quality of Life Outcomes

While considerable attention has been given to global QoL outcomes, other HRQL outcomes are highly relevant to older HNC patients and worth exploring in more detail. As with global QoL, most prospective studies fail to demonstrate consistent age-related differences in other self-reported HRQL outcomes (73–80), with some differences attributable to normal aging, irrespective of whether the patient has cancer.

In a prospective study of HNC patients from Sweden and Norway, Hammerlid et al. reported worse baseline (at the time of diagnosis) scores for older patients (≥75 years) on multiple scales/items of the QLQ-C30 and H&N35; however, these differences were only clinically relevant (≥10) for physical functioning, constipation, dyspnea and coughing. Older patients, however, reported better emotional functioning (99). At the 12-month follow-up, older patients (≥65 years) reported better role (difference = 11) and emotional functioning (difference = 10) but more significant problems with sexuality (difference = 18) and sticky saliva (difference = 15) than younger patients (69). Further, older patients reported less significant changes from baseline in role functioning and dryness of mouth. Nonetheless, changes from baseline in the senses (16 vs 10), sexuality (17 vs 4) and nutritional supplements (10 vs 5) were more severe.

In the previously mentioned Swedish study by Berg et al., the authors reported similar or better scores in the older cohorts with the exception of physical functioning, which was uniformly better in younger patients across the study. One exception was at the 3-month assessment. Here, differences in physical functioning were neither clinically nor statistically significant. This is largely explained by more significant declines in younger patients between baseline and 3 months. With further follow-up and recovery, however, the younger group regained its initial advantage. Older patients also reported less appetite loss and financial difficulties at various time points in this study (60). Notably, the oldest patients (≥80 years) reported worse fatigue, role functioning, and feeling ill at 12 months compared to those under 80 years. The Turkish study, which reported better global QoL at the end of RT, also found that older patients fared better with regards to senses problems and reported less weight loss, but scores were similar in other domains (61). Aoki et al., on the other hand, reported significantly worse social wellbeing and more HNC concerns in older patients (>75 years) at 6 months, as assessed by the FACT-H&N. Again, this appeared to be explained by younger patients showing better and ongoing recovery with further follow-up compared to the older patients.

Longitudinal HRQL outcomes for 785 HNC patients were collected in a North Carolina study using the FACT-H&N, with questionnaires administered to newly diagnosed HNC patients who were then assessed again at two subsequent follow-ups (median 22- and 42-months post-treatment, respectively) (70). Like most prospective studies reviewed, this series contained patients with various HNC subsites and disease stages, and they were treated with any combination of surgery (57%) and/or radiation (77%), with many also receiving concurrent chemotherapy (41%). A linear mixed model incorporating time since follow-up was used on each of the wellbeing domains, but not the total score. Age was divided into four categories, <50 years (19%), 50-64 years (49%), 65-74 years (25%) and ≥75 years (7%). Older patients generally fared better, with superior scores across the physical, emotional and functional wellbeing domains and the HNC additional concerns subscale. No significant differences were observed on the social wellbeing domain.

Physical functioning has also been reported as worse in older patients in other series, sometimes as an isolated finding among all available items/scales/domains (63), or in conjunction with limited other differences (76). While many of these studies suggest that older patients are not more adversely affected, some studies, such as that by Van Der Schroeff et al. have shown worse outcomes (72). In this longitudinal study, 118 older (≥70 years) and 148 younger (45-60 years) patients were followed for three to six years. The QoL component of the study was limited to those who completed all HRQL questionnaires (n=24 and n=33 patients, respectively). Again, the study sample was quite heterogenous in terms of tumor site, disease stage and treatment received. In the QoL cohort, about half the patients had oral cavity cancers. Patients were enrolled at diagnosis and there were no differences in the baseline EORTC QLQ-C30 and H&N35 scores. A more significant decline in physical functioning was seen in the older patients by 12 months post-treatment and this difference persisted at follow-up (i.e. three to six years post-treatment). The older patients also tended to report worse HNC symptoms, including statistically and clinically significant worse social eating (43 vs 23), swallowing (32 v 19) and speech (30 vs 19) at 12 months. Score differences on the swallowing and speech measures remained statistically significantly and clinically worse in the older patients with longer follow-up.

### Laryngectomy Patients

Singer et al. conducted a prospective study of patients undergoing laryngectomy in eight German centers (34). HRQL data was collected at prior to undergoing a laryngectomy, just before hospital discharge, during inpatient rehabilitation, and 12 months later (EORTC QLQ-C30 and QLQ-H&N35). In this study, 101/175 (58%) patients were aged <60 years, 51/174 (29%) were aged 60-69 years, and 22/174 (13%) were ≥70 years. A multivariable regression analysis was undertaken to explore variations in HRQL changes from baseline to 12 months. While several outcomes, such as physical, role and social functioning, and some of the symptom items from both the QLQ-C30 and H&N35 were significantly worse one year after laryngectomy, age (analyzed as a continuous variable) was not a significant ‘predictor of scale/item scores’. On the other hand, Woodard et al. conducted a cross-sectional study in laryngectomy survivors using the Head and Neck Cancer Inventory (HNCI). Analysis of HNCI scores was limited to 33/58 survivors at median follow-up of 37 months post laryngectomy. The study included patients who had received primary surgical treatment and those who had received salvage surgery, however, the final patient contributions to the HRQL data was not reported. Younger patients (<65 years, 15/33) fared far worse across all the functional and attitude domains, as well as the global QoL score (54 vs 75). Those patients needing a laryngectomy as a component of their cancer treatment represent a distinct survivorship cohort amongst HNC patients, one resulting in many HRQL challenges, including loss of communication, body image changes and, potentially, loss of functional employment. While all patients will no doubt struggle with the physical and psychological impacts of a laryngectomy, younger patients in the prime of their adult lives may be especially vulnerable to the consequence of this life changing event.

### Comparison to Age- and Gender-Matched Populations

Several studies have contrasted the outcomes of elderly patients to age- and gender-matched cohorts to differentiate HRQL changes attributable to treatment vulnerability and those that may be expected as a consequence of aging alone. Ferri et al. reported the outcomes of patients aged >75 years who had undergone HNC surgery with microsurgical reconstruction. This multi-site cross-sectional study used the SF-36 to assess HRQL two years after surgery. Of the eligible patients, 76/115 had died, leaving 39/115 enrollees with an average age of 81 years at the time of surgery. Most patients had also received adjuvant treatment (26/39, 67%). While the Mental Component Summary scores were similar to an age-matched Italian cohort, the average Physical Component Summary scores for the treated cohort indicated *better* physical health (46.5 vs 37.9) compared to the age-matched population. In a series of 50 older patients (>65 at time of treatment) who were at least one year from curative-intent radiotherapy (definitive or adjuvant), Ruhle et al. contrasted cross-sectional HRQL outcomes to an age-matched German sample (30). Using the EORTC QLQ-C30, patients reported very similar global QoL to the matched group. However, social functioning (85 vs 71), appetite loss (8 vs 19) and constipation (7 vs 20) were worse in the treated group; conversely, pain scores were lower (22 vs 33).

In a larger study also from Germany, 817 patients were enrolled to a cross-sectional study which compared HRQL, emotional distress and fatigue in HNC patients to a gender- and aged-matched population (<65 years, n=476; and ≥65 years, n=341) (100). The study sample included all HNC subtypes and a mix of primary and adjuvant treatment. Younger treated patients were more adversely affected (larger negative impacts) compared to their age- and gender-matched peers across all measured outcomes. Even so, older patients (≥65 years) did report worse HRQL compared to their matched peers. Global QoL (EORTC QLQ-C30) was worse for both the older males (65 vs 54) and females (63 vs 54). Older patients also reported worse fatigue (males 45 vs 24, females 48 vs 25) compared to the non-cancer patients.

## Psychosocial Distress

The term “distress”, in the context of cancer, was chosen by the NCCN because it is felt to be less stigmatizing than descriptors like “psychiatric” and “psychosocial” concerns. It is defined by the NCCN as “a multifactorial, unpleasant, emotional experience of a psychological (cognitive, behavioral, emotional), social, and/or spiritual nature that may interfere with the ability to cope effectively with cancer, its physical symptoms, and its treatment (101)”. It encompasses both normal feelings such as vulnerability, sadness, and fears, as well as problems that can significantly impact a patient’s life such as depression, anxiety, panic, social isolation, and personal crisis (101).

This report has focused on multiple types of distress, including the more commonly appreciated forms anxiety, depression and suicidality, but also other measures including fear of cancer recurrence, post-traumatic stress disorder, coping strategies and body image distress. This section will also discuss what is known about pain, fatigue, and sleep in older patients with HNC. Where possible, this review has focused on longitudinal, prospective studies, but this was not always possible for some of the less frequently studied outcomes, and where cross-sectional studies have provided novel or additional information, they have been included. The variation in instrument selection in these reports is considerable, and as for the HRQL section, cross-comparison is limited by significant variations in their content and application.

### Instruments Measuring Psychosocial Distress in HNC

Tools commonly used to measure distress in HNC research are listed in **Table 7**. While most HRQL measures include mental health and/or wellbeing scales, this section will focus on tools specifically designed to assess anxiety, depression and/or distress. These include: (1) generic measures which may also be used in a non-cancer population, such as the Geriatric Depression Scale (GDS), Hospital Anxiety and Depression Scale (HADS), Beck Depression Inventory (BDI), and Patient Health Questionnaire 9 (PHQ-9); (2) generic cancer measures, such as the Distress Thermometer (DT), Mini-Mental Adjustment to Cancer (Mini-MAC), and Distress Inventory for Cancer (DIC2); and (3) head and neck cancer specific instruments, such as the Patient Concerns Inventory (PCI). Additionally, there are tools for assessing other factors that directly impact patient distress levels, such as coping style, fear of cancer recurrence, fatigue and sleep, social support, pain, and body image. These will be discussed in their respective sections.

**Table 7:** Common Instruments used to capture distress in HNC studies.

Instrument	Domains covered	Validation cohort age range	Recommended Age use
***Generic***			
Geriatric Depression Scale (GDS) (102)	Depression	General elderly population	≥65 years
Hospital Anxiety and Depression Scale (HADS) (103)	Anxiety and depression	General medical population	≥18 years
Patient Reported Outcomes Measurement Information System (PROMIS) (104)	Anxiety and Depression	Patients with chronic conditions	≥18 years
Beck Depression Inventory (BDI) (105)	Depression	Psychiatric & normal populations, ≥13 years	13-80 years
Center for Epidemiological Studies Depression (CES-D) Scale (106)	Depression	General population, ≥18 years	≥18 years
State-Trait Anxiety Inventory (STAI) (107)	Anxiety about an event and as a personal trait	General population	≥18 years
Hamilton Depression Rating Scale (HAM-D) (108)	Depression	Hospital inpatients ≥18 years	≥18 years
Mini-International Neuropsychiatric Interview (MINI) (109)	Major psychiatric disorders	General population	≥18 years
Quick Inventory of Depressive Symptomatology-Self Report (QIDS-SR) (110)	Depression	General population	≥18 years
Coping Orientation to Problems Experienced (COPE) questionnaire (111)	Assesses ways in which people respond to stress	Has been validated in various stressed populations	≥15 years
Patient Health Questionnaire 9 (PHQ-9) (112)	Depression	General population	≥12 years
Brief Illness Perception Questionnaire (B-IPQ) (113)	Cognitive illness representations: consequences, timeline, personal control, treatment control, concern, emotions, and comprehensibility	Chronically ill patients, age not listed	≥8 years
Acute Stress Disorder Inventory (ASDI) (114)	Screening instrument to identify acute trauma	Trauma populations	≥18 years
***Cancer-Specific***			
Distress Thermometer (DT) (115)	Psychological distress	Adult cancer patients	≥18 years
Mini - Mental Adjustment to Cancer (Mini-MAC) (116)	Fighting spirit, positive redefinition, helplessness – hopelessness, anxious preoccupation	Cancer patients aged 18-75 years	≥18 years
Distress Inventory for Cancer (DIC2) (117)	Psychological distress	Cancer patients aged 18-88 years	≥18 years
***head and Neck Cancer-specific***			
Patient Concerns Inventory (PCI) (118)	Physical and functional wellbeing, treatment-related issues, social wellbeing, psychological wellbeing	HNC patients, ≥18 years	≥18 years

### Anxiety and Depression

Patients receiving HNC therapy self-report high levels of psychological distress, depression, and anxiety (119). Despite this awareness, psychological issues often remain unidentified or unassessed, albeit with significant effects on quality of life, functional status, and survival (8). Social isolation and lack of support have been linked to higher cancer mortality rates and poorer treatment tolerance (120). This is especially concerning in an older patient population because many elderly patients live alone (121). Literature on interventions to improve QOL and mood in patients with HNSCC have been summarized elsewhere (122).

Many studies have explored the relationship between age, and anxiety and depression in HNC patients. Due to the sheer volume of published studies, this paper will focus on results from prospective longitudinal studies (**Table 8**); results from cross-sectional studies are largely compatible. The overwhelming majority of prospective studies indicate that older HNC patients experience either similar rates of anxiety and depression (72, 74, 141–156), or less of one or both (123–138), than do their younger counterparts. Results from very few studies indicate worse distress among older HNC patients (139, 140).

**Table 8:** Prospective studies reporting distress and age in elderly patients with HNSCC.

Author	Year	n	Location	Age	H&N subsite	Treatment	Instruments	Study design	Findings
***Measures favoring older***									
Cash et al. (123)	2018	55	USA	Median 58.5 years; range 24-82 years	Mixed	Mixed	PHQ-9	Prospective: baseline	Increases in cognitive/affective symptoms correlated with younger age (age as continuous variable) (p=.012)
Chen et al. (124)	2009	40	USA	Median 55 years (38-90 years)	Non-metastatic HNSCC	Had to include RT (definitive or adjuvant)	BDI-II HADS-Depression	Prospective: baseline, last day of RT, & first follow-up visit	Age <55y significantly associated with post-RT depression on both scales; levels rose during RT & remained elevated at first follow-up visit (p=<.05)
D’Souza et al. (125)	2013	96	Canada	Median ~59 years	Stage III-IV HNSCC	Mixed	HADS	Prospective	Depression significantly associated with age (p=0.04), with younger pts having higher levels of depression
Ghazali et al. (126)	2017	261	United Kingdom	Median 63 years	Mixed Post-treatment NED	Mixed	DT UWQOL	Prospective: at least 6w post-treatment	Higher distress rates for those <55y (p=0.04)
Hammerlid et al. (127)	1999	357	Norway Sweden	Mean 63 years (18-88 years)	Mixed	Mixed	HADS	Prospective multicenter: baseline and 1, 2, 3, 6, & 12m	Patients under 65y scored higher on mental distress scale at diagnosis and 1y than patients >65
Horney et al. (128)	2010	103	United Kingdom	Mean 63 years	Mixed	Mixed	HADS LOT-R Brief COPE SF-12 v2	Prospective: baseline	Younger age significantly associated with Pre-treatment anxiety & negative coping styles (p=.0001)
Howren et al. (129)	2010	306	USA	Mean 60 years	Mixed	Mixed	BDI HNCI	Prospective: baseline, 3m, & 12m	Younger age predicted worse social disruption at 3m follow-up
Humphris and Rogers (130)	2004	87	United Kingdom	Mean 58 years	Mixed	Mixed	HADS CWS	Prospective: at 3, 7, 11, 15m	Patients who smoked consistently through the study period were significantly younger (by 8y on average) & had higher levels of distress following treatment
Ichikura et al. (131)	2016	117	Japan	55.6% were >65	Mixed All hospitalized	Mixed	HADS FACT-HN	Prospective	Age </=65 associated with higher levels of distress at time of hospital admission (p=.03)
Kanatas et al. (132)	2012	204	United Kingdom	Mean 62 years	Mixed Post-treatment NED	Mixed	UW-QOL PCI	Prospective: at least 6w post-treatment	Age</= 64 more likely to report anxiety/depression (p=.02)
Kumar et al. (133)	2018	75	India	<30 years (n=3), 30-60 years (n=46), >60 years (n=26)	Oral cavity	Mixed (only 7 did not have surgery)	DASS-21 HADS	Prospective: baseline, 1m postop, 3m after discharge	Depression scores at diagnosis higher in those 30-60 (vs >60)
Neilson et al. (134)	2013	101	Australia	Mean 63 years (37-85 years)	Mixed	Mixed	HADS	Prospective: baseline & 3w & 18m after treatment completion	Anxiety scores higher for younger patients
Joseph et al. (135)	2013	220	United Kingdom	Mean 59.5 years	Mixed	Had to include RT (definitive or adjuvant)	HADS LENT-SOMA	Prospective: baseline, post-treatment	Younger age associated with higher anxiety before & upon completion of treatment (p=.002 &.004)
Tang et al. (136)	2011	164	Taiwan	Mean 50.7 years	Mixed cancer types; 35 HNC patients	Mixed	SDS	Prospective	Distress before & after radiation increased more for age 20-39 than age 40-49 and 60-69.
Van Beek et al. (137)	2020	345	The Netherlands	Mean 61 years (36-85 years)	Mixed	Definitive RT +/- chemo	HADS EORTC QLQ-C30 &-H&N35	Prospective: baseline 6w, 3m, 6m, 12m, 18m, 24m	Younger patients had a poorer course of anxiety than older, especially between 12m & 24m follow-up (p=.027)
Wang et al. (138)	2019	211	China	Mean 62 years	Laryngeal ca scheduled for total or partial laryngectomy	Surgery	HADS	Prospective: preoperative	Youngest age group had highest HADS score (p=0.049)
***Measures favoring younger***									
Mccaffrey et al. (139)	2007	24	USA	Mean 73 years (49-82 years)	Mixed (all stage III/IV)	Mixed	SCID DRS	Prospective	Patients >65y experienced more depression than younger pts (p<.04)
Wang et al. (140)	2021	232	China	Median 51 years	Newly diagnosed NPC	RT +/- chemo	HADS	Prospective: Baseline, treatment week 4, post-treatment	Incidence of anxiety & depression in age >40y significantly higher than those <40y at all time points (p=.03 & <.001)
***Age not impacting measures***									
Astrup et al. (141)	2015	207	Norway	Mean 61 years	Mixed	Mixed, but all included RT	CES-D GSDS LFS BPI	Prospective: baseline, 1m, 2m, 3m, 6m	No association between age and depressive symptoms
Bjordal and Kaasa (142)	1995	204	Norway	Mean 67 years	Mixed site, at least 12m post-treatment	All received RT	GHQ-20 EORTC QLQ-C30	Prospective (treated on two RCTs)	No association between distress and age
Bozec et al. (74)	2018	58	France	Evaluated by age </> 65	Oropharynx	Surgery with radial forearm free-flap reconstruction +/- adjuvant RT/chemo	HADS QLQ-C30 & H&N35 VHI DOSS	Prospective: baseline, at least 1y after surgery	Depression & associated psychological states unrelated to age
Chen et al. (143)	2018	133	USA	Median 56 years (21-97 years)	HNSCC, at least 1y disease-free	Mixed	UW-QOL	Prospective: baseline	No correlation between prevalence of depression or anxiety with age
De Leeuw et al. (144)	2000	155	The Netherlands	Mean 59 years	Oral cavity & larynx	Surgery &/or RT, curative intent	SSLI ISSB SPS UCL CLCS CES-D EORTC QLQ C30+3	Prospective: baseline, 6m, 12m	Age was not a predictor of depressive symptoms
Deng et al. (145)	2014	356	China	Mean 46.7 years	Nasopharyngeal	RT or CRT - definitive	DT HADS	Cross-sectional (n=295) & prospective (n=61)	No relationship between distress & age
Derks et al. (146)	2005	183	The Netherlands	“Older” (>/=70 years, n=78); “younger” (45-60 years, n=105)	Oral cavity Pharynx (stage II-IV) or larynx (stage III-IV)	Mixed	EORTC-QLQ-C30 CES-D UCL CLCS	Prospective: baseline, 6m, 12m	No differences in depressive symptoms after treatment
Derks et al. (65)	2004	121	The Netherlands	51 “Older” (n=51); “younger” (n=70) as defined above	Oral cavity, pharynx (stage II-IV) or larynx (stage III-IV)	Mixed	Semi-structured interview with structured & open questions CES-D RSS-12	Prospective: baseline, 1y	No differences in depressive symptoms between groups
Henry et al. (147)	2019	219	Canada	Mean 63 years (30-101 years)	Mixed	Mixed	HADS	Prospective: baseline, 3m, 6m, 12m	No correlation of anxiety or depression with age
Kim et al. (148)	2016	241	Korea	Median 61 years	Mixed	Mixed	BDI EORTC QLQ-C30 & -H&N35	Prospective: baseline	No difference in median age of depressive vs non-depressive patients
Kobayashi et al. (149)	2008	58	Japan	Mean 62 years	Mixed subsite; included new diagnoses & recurrences	Surgery	HADS RSE scale	Prospective: baseline, 7-10d post-op, 6m	No significant difference in self-esteem by age
Kugaya et al. (150)	2000	107	Japan	Mean 61 years	Mixed (oral cavity, pharynx, larynx)	Mostly surgery	Psychological or psychiatric interview HADS	Prospective: baseline	Mean age of patients with & without distress not different
Kunz et al. (151)	2021	120	Germany	Mean 62.6 years (range 41-85 years)	Mixed	Mixed	DT	Prospective: baseline	Distress did not correlate with age
Neilson et al. (152)	2010	75	Australia	Mean 62.5 years (37-85 years)	Mixed	Mixed but all involved RT	HADS FACT-H&N	Prospective: baseline, ~3w post-treatment	No significant association between age & post-treatment distress or anxiety
Panwar et al. (153)	2018	125	USA	Mean 63 years	Mixed	Mixed	QIDS-SR	Prospective	No correlation of depressive symptoms with age
Schell et al. (154)	2018	100	Germany	Mean 64.4 years	Oral HNSCC prior to surgical intervention	Surgery	DT	Prospective: baseline (preop)	Distress score not correlated with age
Speksnijder et al. (155)	2021	141	The Netherlands	Mean 65.6 years	Primary oral HNSCC	Mixed (most surgery)	CES-D	Prospective; multi-center cohort study	Age did not significantly contribute to depression.
Van der schroeff et al. (72)	2006	266	The Netherlands	Not stated	Mixed	Mixed	QLQ-C30 & -HN35 CES-D	Prospective: baseline, up to 6y	No difference in depressive symptoms between the two age groups 3-6y after start of treatment (45-60y vs >/-70y)
Verdonck-de leeuw et al. (156)	2009	55	The Netherlands	Mean 63 years	Mixed	Mixed	HADS QLQ-C30 & -H&N35	Prospective: baseline, first follow-up visit	At diagnosis and follow-up, age not related to distress

BDI-II, Beck Depression Inventory-II; BPI, Brief Pain Inventory; Brief COPE, Brief Coping Orientation to Problems Experienced Inventory; CES-D, Center for Epidemiological Studies Depression; CLCS, Cancer Locus of Control Scale; CWS, Cancer Worry Scale; DASS-21, Depression Anxiety Stress Scales-21; DT, Distress Thermometer; DOSS, Dysphagia Outcome and Severity Scale; FACT-HN, Functional Assessment of Cancer Therapy-Head and Neck module; GHQ-20, General Health Questionnaire-20; GSDS, General Sleep Disturbance Scale;HADS, Hospital Anxiety and Depression Scale; HNCI, Head and Neck Cancer Inventory; ISSB, Inventory of Socially Supportive Behaviors; LENT-SOMA, Late Effects Normal Tissues-Subjective, Objective, Management, Analytic; LFS, Lee Fatigue Scale; LOT-R, Life Orientation Test-Revised; MDRS, Mattis Dementia Rating Scale; PCI, Patient Concerns Inventory; PHQ-9, Patient Health Questionnaire-9; QIDS-SR, Quick Inventory of Depressive Symptomatology-Self Report; QLQ-C30, European Organization for Research and Treatment of Cancer Quality of Life Questionnaire-Core 30; QLQ-H&N35, European Organization for Research and Treatment of Cancer Quality of Life Questionnaire-Head and Neck module; RSE scale, Rosenberg Self-Esteem scale; RSS-12, Reflux Symptom Score-12; SCID, Structured Clinical Interview for DSM-IV; SDS, Symptom Distress Scale; SF-12 v2, Short Form 12 item (version 2) Health Survey; SSLI, Social Support List Interactions; SPS, Social Provisions Scale; UCL, Utrecht Coping List; UWQOL, University of Washington Quality of Life Questionnaire; VHI, Voice Handicap Index.

The largest prospective study to investigate the relationship between distress and age was reported by Hammerlid et al. in 1999 (127). The HADS was used to provide a measure of distress at baseline (after diagnosis but prior to initiating treatment) and one, two, three, six and 12 months after initiation of treatment in a mixed group of 357 HNC patients receiving various therapies. On average, patients <65 years reported higher levels of distress at diagnosis and at the one year follow up than did older patients. Another large prospective study evaluated a mixed group of 306 HNC patients from the US at their initial clinic appointment, and three- and twelve-months post-diagnosis, using the BDI and the Head and Neck Cancer Inventory. Younger age was associated with worse social disruption at three months (129). Prospective studies showing an association between distress and younger patient age have been reported from investigators in the UK, Japan, India, Australia, Taiwan, China, and the Netherlands (131, 133, 134, 136–138). In several studies, younger age is specifically linked to higher rates of anxiety, especially before and during treatment, which some speculate is due to increased fear of recurrence (126, 134, 135, 137).

There are only two prospective studies wherein older patients fared worse with regards to distress, and it is notable that the larger of the two reported on only nasopharyngeal cancer patients in China, with a median age of 51 years. In this study, rates of anxiety and depression in patients over the age of 40 was significantly higher than in those under 40 years at all timepoints; however, the interquartile range was 40-57, and this study is not representative of a broad elderly HNC population. The other prospective study reporting lower distress in younger patients is a very small study from the US reporting on preoperative distress in locally advanced HNC patients. Patients over 65 years were found to experience more depression than younger patients. This study did not utilize commonly utilized instruments and is far too small to draw any useful conclusions (139).

In summary, the preponderance of prospective evidence from a wide range of countries and across most head and neck disease sites and treatments suggests that elderly patients actually fare better with regards to distress as compared to their younger counterparts and that the reasoning for this is likely nuanced. Factors may include mid-life responsibilities more characteristic of younger patients, such as employment, childcare, greater perceived importance of social life, and less earned resilience. As discussed in the following sections, fear of cancer recurrence, pain, fatigue, sleep, body image issues, and post-traumatic stress may also be higher in younger patients. While most of these studies consist of self-reports based on screening tools rather than diagnostic interviews, which is a limitation, the results align with those from the general population; namely, that older people typically report lower levels of distress and anxiety than do younger people (157).

### Fear of Cancer Recurrence

Fear of cancer recurrence (FCR) is a common concern reported by cancer survivors, including patients treated for HNC (**Table 9**). There are a number of methods used to gather information about FCR. FCR severity may be measured using standardized tools like the Fear of Cancer Recurrence Inventory (169, 170) or the Fear of Recurrence questionnaire (166). Concerns about FCR may also be gathered as part of various patient concerns inventories. Irrespective of the method used to gather information on FCR, older cancer survivors report lower levels/rates of FCR on average than younger patients (171). This observation has also been seen in HNC studies where variations in FCR have been analyzed by age (**Table 9**), with the majority showing an increased vulnerability to FCR in younger patients (158–164).

**Table 9:** Head and neck cancer studies reporting variations in fear of cancer recurrence measures by age.

Author	Year	n	Location	Handling of age	H&N subsite	Treatment	FCR instrument	Study design	Findings
**FCR favoring older patients**									
Mirosevic et al. (158)	2019	216	Slovenia, Netherlands	Continuous (range 37-85 years)	OCC, 30% OPC, 34% HPC, 8% LC, 25% CUP, 2%	Pre treatment	CWS	Baseline data from a cross-sectional sub study of NET-QUBIC included psychiatric interviews	Younger patients reported higher FCR (β = .203, *p*<0.001)
Casswell et al. (159)	2021	136	Australia	Continuous (range 42-87 years)	All HPV+ OPC	All RT/CRT	FCRI-SF	Cross-sectional: time since treatment mean 2.97y (range 1.0-5.1y)	Younger age worse on UVA (-0.2/year increase, *p*=0.036)
Rogers et al. (160)	2021	288	United Kingdom	<55 vs 55-64 vs 65-74 vs ≥75 years	OCC, 47% OPC, 32% LC, 14% Other, 8%	Sx alone, 40% Sx +aRT/CRT, 40% CRT/RT, 20%	UW-QoL 5-response item	Cluster control study	Patients <65 more likely to answer more severe FCR (especially younger females <55)
Ghazali et al. (161)	2013	189	United Kingdom	<55 vs 55-64 vs ≥65-74 vs ≥75 years	OCC, 73% Pharyngeal, 23%	Sx alone, 59% Sx + aRT, 32% RT/CRT, 10%	PCI FoR questionnaire	Prospective: post treatment (convenience sample, first appt not always patient’s actual first appointment post treatment completion).	*For first visit:* overall FoR score higher in younger patients and significant FoR in at least one item higher in younger patients *Longitudinal assessment:* About one-third <65 unlikely to encounter significant FoR issues compared to three-quarters of patients ≥65.
Kanatas et al. (162)	2015	813	United Kingdom	Continuous and <65 years	OCC, 48% OPC, 24% LC, 17% Other, 9% CUP, <1%	Sx alone, 49% Sx +aRT, 34% CRT/RT, 14% Unknown, 3%	HNC-PCI	Analysed patients selecting FCR item on PCI	Patients selecting FCR were 4-6 years younger; When analysis first time (n=813) PCI: Percentage ≥65y: X^2^ = 33.1, p< 0.001 and ≥75y: X^2 =^ 14.4, p= 0.002
Rogers et al. (163)	2016	528	United Kingdom	<55 vs 55-64 vs ≥65 years	OCC, 34% OPC, 35% LC, 20% Other, 8%	Sx alone, 38% Sx + aRT, 40% CRT/RT, 16% Unknown, 3%	UWQoL single item: “fear of the cancer coming back” on 5-point scale, “I have no fear of recurrence” to “I am fearful all the time that my cancer might return and I struggle with this” FoR questionnaire	Cross-sectional:	Younger patients reported more severe levels of FoR
Rogers et al. (164)	2015	483	United Kingdom	<55 – 22% 55-64 – 35% 65-69 – 13% 70-74 – 14% ≥75 – 16%	OCC, 57% OPC, 21% Other, 20%	Sx alone, 51% Sx + aRT, 35% RT, 10%	HNC-PCI	PCI-HN data from follow-up clinics collected from 6 different studies, not consecutive; number of responses ranged from 1-≥4	On the PCI, being elderly correlated with fewer items being selected from the psychological, emotional and spiritual wellbeing domain; selecting fear of cancer coming back item reduced with age: 46% vs 42% vs 31% vs 26% vs 20%
Rylands et al. (165)	2016	448	United Kingdom	Continuous	OCC, 40% OPC, 38% LC, 22%	Sx alone, 43% Sx + aRT, 42% CRT/RT, 15%	FoR questionnaire	Cross-sectional	Age was inversely correlated with FoR results
**FCR not impacted by age**									
Rogers et al. (166)	2010	191	United Kingdom	NR	OCC, 72% OPC, 21% Other, 7%	Sx alone, 57% Sx + aRT, 37% RT, 7%	HNC-PCI completed at clinic attendance FoR questionnaire	Cross-sectional: ≥ 6weeks following completion of treatment Two cohorts: PCI plus UWQoL; PCI plus UwQoL & FoR	Non-significant trend in younger patients selecting FoR item on PCI in cohort 1 (analysis of multi-item questionnaire NR)
Llewellyn et al. (167)	2008	55	United Kingdom	Continuous (range 23-89)	Subsites NR	NR	‘Over the past month, how often have you worried about the possibility that cancer might come back?’ Response on 1-5 Likert scale (none of the time to all the time)	Pretreatment and 6-8 month post treatment Examined longitudinal predictors of FCR in survivors of HNC using Leventhal’s CSM as a framework,	FCR was not related to socio-demographic factors including age
Van Liew et al. (168)	2014	138	USA	Continuous	NR (only early vs late stage)	Sx alone, 38% RT only, 11% Combination, 42% Unknown, 9%	FCRI	Cross-sectional	Current age and age at diagnosis were not associated with FCR

aRT, adjuvant radiotherapy; CSM, common sense model CUP, carcinoma unknown primary; CWS, Cancer Worry Scale; FCR, fear of cancer recurrence;FCRI, Fear of Cancer Recurrence Inventory; FoR, Fear of Recurrence; HNC, Head and Neck Cancer; HPC, hypopharyngeal cancer; HPV, human papillomavirus; LC, laryngeal cancer; NR, not recorded; OCSCC, oral cavity cancer; OPC, oropharyngeal cancer; PCI, Patient Concerns Inventory RT/CRT, radiotherapy/chemoradiotherapy; SF, short form; Sx, surgery; UVA, univariate/variable analysis; UW-QoL, University of Washington Quality of Life questionnaire.

Concerns about cancer recurrence (perhaps fear of progression) may begin early on the cancer journey, and age-related differences may be observed even before treatment has commenced. Mirosevic et al. reported FCR outcomes in 216 newly diagnosed HNC patients using the 8-item Cancer Worry Scale (158). FCR scores were higher in younger patients, and age, along with baseline HADS anxiety scores were the strongest predictors of baseline FCR. As shown in **Table 9**, many studies have included substantial proportions of patients treated for oral cavity cancer with surgery alone. Irrespective of study design, younger patients appear more vulnerable, with the oldest patients (≥75 years) reporting low or lower rates of FCR (162, 164). In a cross-sectional cohort of 136 patients with HPV-associated oropharyngeal cancer treated with definitive (chemo)radiotherapy Casswell et al. measured FCR using the FCRI-short form (which assesses the severity domain of the larger FCRI version). In this study, age was analyzed as a continuous variable, and it is interesting to note that even with this favorable HNC cohort harboring a typically low risk of recurrence (given most had had a complete response to treatment and the mean time since treatment completion was 3 years) that older patients continued to report lower rates of FCR.

### Body Image Distress in Older Patients With HNSCC

Body image studies in HNC have mostly been undertaken in samples of patients undergoing major surgery for their cancer, as detailed in **Table 10**. In summary, evidence of an association between age and body image distress is weak but limited cross-sectional and retrospective data suggest similar or less severe body image distress in older HNC patients (172–175). Further high-quality research is needed in this area.

**Table 10:** Studies reporting on body image, pain, fatigue/sleep, and post-traumatic stress.

Author	Year	No.	Location	Age	H&N Subsite	Treatment	Instrument	Study Design	Findings
***Body Image***									
Chen et al. (172)	2020	154	China	Mostly men, 40-65 years	Mostly thyroid, oral cavity, larynx	Surgery within past month	BIS HADS PSSS SCSQ	Cross-sectional	Younger patients (<40y) had significantly higher BIS score (more dissatisfaction), which is associated with higher anxiety.
Clarke et al. (173)	2014	49	United Kingdom	Mean 60.4 years	HNSCC (stage III-IV, recurrent, or radiation failure)	Mixed	DAS24 HADS	Cross-sectional: baseline and 9m	No correlation between age & DAS-24 score
Vilaseca et al. (174)	2006	49	USA	Mean 62.7 years (44-82 years)	Larynx	Total laryngectomy	UW-QOL	Cross-sectional	Younger patients more likely to express dissatisfaction with appearance
Zebolsky et al. (175)	2021	103	USA	Median 66 years	Mixed, mostly oral cavity and cutaneous	All involved microvascular reconstruction	ARPD SF scales of the FACE-Q	Retrospective: median time of follow-up at time survey completed 13.5m	Age not significantly associated with ARPD or SF scores
***Pain***									
Cramer et al. (176)	2018	175	USA	Median 65 years	HNC survivors >/=1 y after diagnosis	Mixed	Pain and QOL assessed with multiple instruments	Retrospective	Patients <55y more likely than older patients to develop pain (odds ratio of 0.38 for 75+ vs <55)
Moye (177)	2014	170	USA	Mean 64.6 years (27-88 years)	HNC, esophageal, gastric, or colorectal cancer from the VA (40% H&N)	Mixed	PROMIS PHQ-9	Prospective: 6m, 12m, & 18m	At 6m post-diagnosis, younger adults described higher levels of pain & of pain intensity.
Hassanein et al. (178)	2001	68	United Kingdom	Median 58 years	Oral cavity, oropharyngeal, maxillary sinus cancer, 6m to 6y post-treatment	Mixed	UW-QOL HADS MAC-Q SSQ6	Retrospective	Young pts (≤60) had more functional problems, highly significant for swallowing & shoulder pain, and significant for pain.
***Fatigue/Sleep***									
Hickok (179)	2005	372	USA	Mean age of HNC patients 61 years	Receiving RT for cancer (HNC, n=23)	RT	In-house 12-item symptom inventory	Prospective: inventory completed weekly for 5 weeks during RT	Age not predictive of fatigue severity at any time point
Rogers et al. (180)	2008	58	USA	Mean 60 years	Mixed	Mixed	FSI PSQI FACT-Cog	Cross-sectional	Fatigue, sleep dysfunction, poorer cognitive function all associated with younger age (all statistically significant)
Santoso et al. (181)	2020	560	The Netherlands	Mean 63 years	Mixed, patients on prospective NET-QUBIC study	Mixed	PSQI	Cross-sectional, multi-center	Younger age significantly associated with poor sleep (p=.049)
***PTSD***									
Holtmaat et al. (182)	2016	74	The Netherlands	Median 61.2 years (41-83 years)	HNC survivors with psychological distress (HADS-Anxiety or -Depression >7)	Mixed	PTGI	Cross-sectional	Age not significantly related to PTGI score
Kangas et al. (183)	2005	82	United Kingdom	Not stated	New diagnosis of head & neck (n=56) or lung cancer	Mixed	ASDI EORTC QLQ-C30 Mini-MAC PDEQ BDI-II STAI-Y PTCI DUKE-SS	Prospective: assessed for acute stress disorder at baseline, and for PTSD at 6 months post-diagnosis	Incidence of PTSD higher in younger patients (mean age for PTSD 49, vs 61 for no PTSD, p <.05)
Moschopoulou et al. (184)	2018	93	United Kingdom	Mean 66 years	HNC in follow-up stage and at least 2y out from diagnosis	Mixed	HADS ESSI ALTTIQ DAS24 QLQ-C30 PCL-C	Cross-sectional	Negative association between PCL-C score and age (younger age is associated with posttraumatic stress syndrome)
Posluszny et al. (185)	2014	42	USA	Mean 55 years	Newly diagnosed HNC pts	Mixed	PCL-C	Prospective study of dyads (patient plus partner)	Age not related to PCL score
Richardson et al. (186)	2016	91	New Zealand	Not stated	Mixed	Mixed	FACT-H&N GHQ-12 PSS-SR Brief COPE	Prospective; baseline & 6m	Age not significantly correlated with PTSD at diagnosis or follow-up

ALTTIQ, Assessment of Life Threat and Treatment Intensity;ARPD, Appearance-Related Psychosocial Distress; ASDI, Acute Stress Disorder Inventory; BDI-II, Beck Depression Inventory-II; BIS, Body Image Scale; Brief COPE, Brief Coping Orientation to Problems Experienced Inventory DAS24, Derriford Appearance Scale-24; DUKE-SS, Duke-UNC Functional Social Support Scale; ESSI, ENRICHD Social Support Inventory; FACT-Cog, Functional Assessment of Cancer Therapy-Cognitive; FACT-H&N, Functional Assessment of Cancer Therapy-Head and Neck module; FSI, Fatigue Symptom Inventory H&N, head and neck; GHQ-12, General Health Questionnaire-12; HADS, Hospital Anxiety and Depression Scale; HNC, head and neck cancer; HNSCC, head and neck squamous cell carcinoma; MAC-Q, Memory Complaint Questionnaire; Mini-MAC, Mini-Mental Adjustment to Cancer; NET-QUBIC, Netherlands Quality of Life and Biomedical Cohort; PCL-C, PTSD Checklist-Civilian; PDEQ, peritraumatic dissociative experiences questionnaire PHQ-9, Patient Health Questionnaire-9; PROMIS, Patient Reported Outcomes Measurement Information System; PSSS, Perceived Social Support Scale; PSQI, Pittsburgh Sleep Quality Index; PSS-SR, Posttraumatic Symptom Scale Self-Report versionPTCI, Posttraumatic Concerns Inventory; PTGI, Post-Traumatic Growth Inventory; QLQ-C30, European Organization for Research and Treatment of Cancer Quality of Life Questionnaire-Core 30; SCSQ, Simplified Coping Style Questionnaire; SF, Social Functions; SI, Symptom Inventory; SSQ6, Social Support Questionnaire-Short Form; STAI-Y, State-Trait Anxiety Inventory-Y;UW-QOL, University of Washington Quality of Life Questionnaire; VA, Veterans’ Affairs.

### Fatigue and Sleep in Older Patients With HNSCC

There is a paucity of data on sleep quality in HNC patients; however, insight into sleep quality would likely help healthcare providers better support their patients. Sleep disturbance and fatigue have been shown to contribute significantly to depressive symptoms, and sleep is unique as it is an often treatable risk factor (141). Tools used to assess sleep and fatigue in HNC patients include the Pittsburgh Sleep Quality Inventory (PSQI), Fatigue Symptom Inventory, Lee Fatigue Scale, and General Sleep Disturbance Scale (GSDS).

**Table 10** outlines studies reporting on fatigue and sleep in head and neck cancer patients. Hickok et al. prospectively administered a one-page symptom inventory to 372 patients weekly for five weeks during radiotherapy. Of note, this included patients with all types of cancer and only 23 had HNC. Mean age of the HNC patients was 61 years, and age was not predictive of fatigue severity at any time in this study (179). Other studies are cross-sectional in design but have the advantage of being limited to head and neck cancer and, like most of the other domains reviewed, they appear to show an association between younger age and poor sleep quality and fatigue (180, 181). As with body image distress, more high-quality research would be helpful in investigating this relationship further.

### Pain in Older Patients With HNSCC

Pain is experienced as one of the most impactful sources of distress in cancer survivors and has been linked to poorer outcomes and decreased QoL (177). Most of the literature on pain and HNC indicates that younger patients experience higher levels of pain than older patients. Relevant studies are reported in **Table 10**. In summary, one prospective trial conducted using the PROMIS scale in a Veterans Administration Hospital population showed that at six months post-diagnosis, younger adults (64 years and younger) described significantly higher levels of pain and of pain intensity as compared to older adults (≥65). It is notable that this was a mixed population of cancer patients, with 40% having HNC (177).

Two retrospective papers show similar trends, with younger patients experiencing more pain. Interesting findings from these studies include that the incidence of pain among survivors was as high as 45.1% at a median of 6.3 years since treatment completion, and that pain was the third-most important issue identified by survivors, after swallowing and saliva. Additionally, patients with pain were significantly more likely to report issues with anxiey and mood (176). The findings also suggest that younger patinets may have more functional problems, including dysphagia and shoulder function (178).

This is an interesting observation with many possible confounders, and future research efforts may look to focus on understanding this association and strategies to help support younger patients affected by HNC.

### Post-Traumatic Stress in Elderly HNC Patients

As with other factors influencing distress, the literature evaluating the relationship between PTSD and age in HNC patients is mixed, with some showing no difference in rates, and some showing higher incidence in younger patients (**Table 10**). There have been two similarly powered prospective studies, with contradictory findings regarding an asssociation between PTSD and age (183, 186). Cross-sectional studies, as well as one small prospective study of patient-partner dyads, have shown mixed results (182, 184, 185). In summary, the literature suggests either no association between age and PTSD, or a trend toward higher rates in younger patients.

### Differences in Coping Styles

Coping refers to the ways in which people respond to and behave during stressful events, and it is likely to influence how one evaluates his or her quality of life (146). Research suggests older people adapt better to cancer because serious illness is more common and expected with increasing age and their experience of physical decline may be less dramatic. Younger people also often have more stage of life-related stressors like child-rearing and employment (146).

Pre- and post-treatment depression and anxiety have been linked to ineffective coping styles such as helplessness/hopelessness, fatalism, and avoidant coping (128). Some authors have noted that although depression and anxiety are more easily measured than coping styles, patient coping strategies may be more amenable to intervention (128). Notably, coping styles seem to vary quite predictably with age in samples of HNC patients (**Table 11**).

**Table 11:** Studies reporting on coping styles.

Author	Year	No.	Location	Handling of Age	H&N subsite	Treatment	Instrument	Study Design	Findings
Aarstad et al. (187)	2012	96	Norway	Mean for twice-interviewed patients 56 years	HNC survivors <80 years old	Mixed	COPE BDI	Cross-sectional	Age not significantly associated with coping style
Aarstad et al. (188)	2011	139	Norway	Mean 60 years	HNC survivors at least 12 months NED <80 years old	Mixed	COPE	Cross-sectional	Age not significantly associated with coping style
Derks et al. (146)	2005	183	The Netherlands	“Older” (≥70 years, n=78) “Younger” (45-60 years, n=105)	Oral cavity, pharynx (stage II-IV) or larynx (stage III-IV)	Mixed	EORTC-QLQ-C30 CES-D UCL CLCS	Prospective; baseline, 6m, 12m	Older & younger patients use different coping & locus of control strategies. Younger patients used more active coping strategies & perceived more internal control. Older patients tended to use religion.
Horney et al. (128)	2010	103	United Kingdom	Mean 63 years	Mixed	Mixed	HADS LOT-R Brief COPE SF-12 v2	Cross-sectional	Younger age significantly associated with negative coping styles (“helplessness”, denial, venting, substance abuse, behavioral disengagement)
Ichikura et al. (189)	2017	116	Japan	Not stated	HNC outpatient survivors who had completed treatment and were >20 years old	Mixed	Brief-COPE BDI-II	Cross-sectional	Age >/=65 associated with “resigned” coping style (vs dependent or problem-focused)
Verdonck-de leeuw et al. (190)	2007	41	The Netherlands	Mean 59 years	Mixed, at follow-up clinic	Mixed	HADS	Cross-sectional, administered to patient-spouse pairs	Total HADS score not significantly related to age but is significantly related to passive coping style (which can be associated with more pessimism, worry, & distress) & non-expression of emotions

BDI-II, Beck Depression Inventory-II; Brief COPE, Brief Coping Orientation to Problems Experienced Inventory;HADS, Hospital Anxiety and Depression Scale; CES-D, Center for Epidemiologic Studies Depression scale; LOT-R, Life Orientation Test-Revised; QLQ-C30, European Organization for Research and Treatment of Cancer Quality of Life Questionnaire-Core 30; SF-12 v2, Short Form 12 item (version 2) Health Survey UCL, Utrecht Coping List; CLCS, Cancer Locus of Control Scale.

One prospective study showed that older and younger patients use different coping and locus of control strategies, with younger patients using more active coping strategies and perceiving more internal control, and older patients tending to use religion. These differences in coping styles did not significantly impact QoL or depressive symptoms, but avoidant coping style was associated with worse depressive symptoms and QoL regardless of age group (146).

One retrospective study showed that younger age is significantly associated pretreatment anxiety and with negative coping styles such as helplessness, denial, venting, substance abuse, and behavioral disengagement (128). Another showed that older patients tended to use a “resigned” coping style (versus dependent or problem-focused). The dependent-coping pattern, which includes smoking, drinking, seeking support and engaging self-distraction, was found to be the most common style and was also the most likely to be associated with depression (189).

There are other studies, in contrast, that have shown no association between age and coping style, most notably, the Norweigan studies by Aarstad et al. (187, 188). Again, this area would benefit from further exploration.

### Suicidality in Older Patients With HNSCC

At the extreme end of unrecognized or poorly managed psychosocial distress are self-inflicted injuries including suicide. Patients with any cancer diagnosis have been reported to have higher risks of suicide (191–193) or non-fatal self-injury (NFSI) compared to the general population, and patients with HNC may harbour even higher rates compared to most other types of cancer (191, 193–196). Other factors such as increasing age, social supports and a history of mental health have also been associated with an increased suicide risk (197).

Zaorsky et al. compared rates of suicide outcomes in cancer patients *via* the US-based SEER database from 1973 to 2014 (191). Compared to the general population, the relative risk of suicide was highest for those patients diagnosed with head and neck, lung, and testicular cancer, as well as Hodgkin lymphoma. The standardized mortality ratio (SMR) for HNC patients was highest in the first year after diagnosis and decreased with time from the initial diagnosis. Across the entire cancer cohort, elderly, white men with localized cancer who were not married were at highest risk. In a SEER study focusing primarily on patients with HNC (including thyroid), Kam et al. reported an adjusted (age, sex, race) suicide rate of 37.9 per 100 000 person-years, corresponding to a SMR of 3.21 (95% CI 2.18-4.23). Across all HNC subsites, older patients (60-79 years and >80 years) had the highest SMR. All of the HNC subsites, except for thyroid, had a higher risk of suicide than the general population, with those patients diagnosed with hypopharynx having the highest risk (SMR 13.91, 95% CI, 11.78-16.03). There was a decline in SMR over time for most of the subsites, though for all but thyroid and nasal cavity/sinuses, the risk exceeded that of the general population, while those with NPC had a persistently elevated and high risk even 15 to 30 years after their diagnosis.

In a Korean study, Choi et al. reported higher suicide risk amongst cancer patients older than 60 years of age compared to sampled population data (includes ~10% of the entire population (193). Similar to the Zaorsky et al. study, the risk of suicide was highest in the first year after any cancer diagnosis, accounting for 34.9% of all cases (median time to suicide was 1.74 years). For those patients diagnosed with a HNC, the adjusted hazard ratio was 2.28 (95% CI 1.47-3.54) which was amongst the highest risk for any cancer type.

## Unmet Needs in Elderly Patients With HNSCC

Although various unmet needs tools are available for use in cancer populations, their content and structure is highly variable. Individual items and needs domains covered may range from physical and psychological needs to informational needs. Needs domains may also include, but are not limited to, nutritional, dental, financial, and sexual needs. Shunmugasundaram et al. recently conducted a systematic review comparing the content of self-reported unmet needs instruments in head and neck cancer populations, concluding that the PCI was the most comprehensive of the available tools, covering all twelve specified needs included in the conceptual framework of unmet needs developed by the research team (198). This is perhaps not surprising given it was the only unmet needs instrument which was developed specifically in a head and neck cancer population and has undergone continual refinement since its initial development. Studies of HNC patients including unmet needs tools are summarized in **Table 12**.

**Table 12:** Studies reporting variations in unmet needs by age in HNC patients.

Author	Location	No.	Age(range)	Head Neck Subsite	Treatment	Instrument	Domains covered	Study design	Assessment Time	Variation – No. needs	Variation - specific needs
Giuliani et al. (199)	Canada	158	Median 64 years (19-89 years)	OC, 29% OPC, 17% HPC/LC, 17% NPC, 5% Other 33%	Sx, 22% Sx + aRT/CRT, 35% RT/CRT, 40%	CaSun	35 items, 5 domains: Existential, Survivorship, Comprehensive Cancer Care, Information, Quality of Life, and Relationships	Cross-sectional	Diagnosis to follow-up (68% ≥6m post treatment)	Older less unmet needs	NR
Henry et al. (200)	Canada	127	Mean 61 years	OC, 35% OPC, 8% LC, 21% Thyroid, 8% Other, 28%	None, 2% Sx alone, 15% Sx + aRT/CRT, 41% RT/CRT, 43%	SCNS-SF34	34 needs items, 5 domains: Psychological, Health system and information, Physical and daily living, Patient care and support, and Sexuality	Cross-sectional	Post-treatment (49% ≥1y)	No differences by age	NR
Henry et al. (201)	Canada	145	Mean 63 years	OC, 16% OPC, 44% HPC, 1% LC, 14% NPC, 3% Other, 14% Unknown, 9%	Any: Sx, 36% RT, 86% CT, 67%	SCNS-SF34	34 needs items, 5 domains: Psychological, Health system and information, Physical and daily living, Patient care and support, and Sexuality Factor analysis determined 3 dimensions: 1. psychological and physical and daily living domain; 2. health system and information, patient care and support domain; and 3. sexuality domain	Prospective: reported post-treatment outcomes	3 months post-treatment	No differences by age	Only the sexuality domain varied by age
Wells et al. (202)	Scotland	319	Mean 65 years (27-91 years)	OCC, 34% LC, 33% OPC, 20% NR, 13%	Sx alone, 27% Majority “combination therapy”	PCI	45 concerns items, no domains *	Cross-sectional postal	Post treatment (69% ≥ 1y)	Younger patients more concerns: (Mean) <45 - 11.5 45-54 – 11.6 55-64 – 10.1 65-74 – 7.6 ≥75 – 7.9	NR
Rogers et al. (164)	United Kingdom	483	Median 63 years (56-72 years)	OCC, 57% OPC, 21% Other, 20%	Sx alone, 51% Sx + aRT, 35% RT, 10%	PCI	57 concerns items; Physical function and wellbeing, Treatment-related, Social care and social wellbeing, Psychological, Emotional and Spiritual wellbeing^a^	PCI data from follow-up clinics collected from 6 different studies, not consecutive; number of responses ranged from 1-≥4	Post treatment	No difference in total concerns	Decrease in FCR from 46% (<55) to 20% (≥75) Older patients more frequently selected bowel habits, constipation, diarrhoea, coughing and memory Older patients selected fewer items in the psychological, emotional and spiritual wellbeing domain
Ghazali et al. (203)	United Kingdom	674	Mean 64 years	OCC, 50% Pharynx, 25% LC, 17% Other, 6%	Sx alone - 50% Sx + aRT, 33% CRT, 14% Unknown, 3%	PCI	57 concerns items; Physical function and wellbeing, Treatment-related, Social care and social wellbeing, Psychological, Emotional and Spiritual wellbeing *	PCI data for first time completers	Post-treatment (Median 32m, range 14-58m)	NR	Older patients less likely to select items from the: 1. psychological, emotional wellbeing and spiritual domain; and 2. Social care and wellbeing domain
O’Brien et al. (204)	Ireland	583	<60 35% 60-69 37% ≥70 27%	OCC, 47% Pharyngeal, 19% LC, 30% Unknown, 4%	Any: Sx, 69% RT, 64% CT, 22%	SCNS-SF34	34 needs items, 5 domains: Psychological, Health system and information, Physical and daily living, Patient care and support, and Sexuality	Cross-sectional	Post-treatment (50% > 5y)	NR	Older patients reported fewer items in each domain
Manne et al. (205)	New Jersey	92	Mean 62 (33-79)	OCC, 62% OPC, 38%	Any: (self-reported) Sx, 66% RT, 84% CT, 65%	Shortened version - SCNS-SF34	Only 25 items, 5 domains: Psychological, Health system and information, Physical and daily living, Patient care and support, and Sexuality	Cross-sectional	Post-treatment	Age not associated with total support needs;	Age not associated with information needs
Chen et al. (206)	Taiwan	112	Mean 53 (27-80)	OCC, 100%	All surgery	Chinese version SCNS-SF34	34 needs items, 5 domains: Psychological, Health system and information, Physical and daily living, Patient care and support, and Sexuality	Cross-sectional	Post-surgery (10-14 days)	Older age associated with decreasing overall needs	Older age associated with decreasing sexual needs, but not other domains
Lee et al. (207)	USA	342	Mean 56	Tongue, 48% Tonsil, 18% Other 30% % OPC, 18%	Any: Sx, 71% RT, 84% CT, 58%	SUNS	89 items, 5 domains: Information, Access and continuity of care, Emotional health, Relationships, and Financial concerns.	Cross-sectional	Post-treatment (>3m)	NR	Older age associated with less relationship and emotional needs

aRT/CRT, adjuvant radiotherapy/chemoradiotherapy; CaSUN, Cancer Survivors’ Unmet Needs Measure; CT, chemotherapy; FCR, fear of cancer recurrence; HPC, hypopharyngeal cancer; LC, laryngeal cancer; NPC, nasopharyngeal cancer; NR, not reported; OCC, oral cavity cancer; OPC, oropharyngeal cancer; PCI, patient concerns inventory; RT/CRT, radiotherapy/chemoradiotherapy; SCNS-SF34, Supportive Care Needs Survey–Short Form; SUNS, Survivors Unmet Needs Survey; Sx, surgery.

a The PCI has evolved over time with additional items and grouping into domains (164, 208).

Almost all of these studies focused on patients who had emerged from the acute post-treatment period with exception of the Canadian study by Giuliani et al. which included patients from diagnosis through to survivorship and that by Chen which evaluated patients shortly after they had undergone surgery (206). The survivors included in many of these studies are quite heterogenous, as has been seen in many of the QoL and psychosocial distress reports with only the study by Chen et al. focusing on patients with oral cavity cancer (206). Despite variability in the instruments used, the available evidence suggests that older patients report fewer unmet needs than younger patients. In those studies which report a summed overall assessment of unmet needs, most have shown a lower burden in older patients (199, 202, 206) or no age-related differences at all (164, 200, 201, 205). For individual domains, older patients generally report less unmet needs, including in the sexuality domain (201, 206), or domains assessing psychological/emotional or spiritual needs (164, 203, 204, 207).

## Novel Approaches

### Biological Age

Recently, Xiao et al. reported associations between epigenetic age acceleration (EAA), survival and HRQL in HNC patients (22). EAA )describes discordance between the chronological age of an individual and their “epigenetic age”; EAA’s can be both positive, where the epigenetic age is greater than the patients’ chronological age, or more simply where aging has been accelerated, and negative where the reverse is seen, indicating a slower aging process. In this sample, they identified clinical variables associated with increased EAA, which was associated with worse survival outcomes. However, they also analyzed longitudinal variations in the FACT-H&N. Using a multivariable generalized estimating equation, and controlling for covariates, patients with a negative EAA had higher FACT-H&N total score over, indicating better QoL, than those with a positive EAA. The estimated score difference was 10.6 points (*p*<0.001), which is in excess of a commonly cited MCID (98). These are intriguing results, but how best to utilize these measures in clinical practice and how to incorporate them into complex treatment decision-making pathways and counselling will require additional research.

### Comprehensive Geriatric Assessment

Across the oncology landscape, interest is increasing in the use of comprehensive geriatric assessments (CGAs) to aid clinical decision making in older individuals with cancer. The CGA has been defined as “a multidimensional, interdisciplinary diagnostic process focusing on determining an older person’s medical, psychosocial, and functional capabilities to develop a coordinated and integrated plan for treatment and long-term follow-up” (209, 210). The International Society of Geriatric Oncology have recommended a core set of domains to assess in a CGA, including functional status, fatigue, medical comorbidities, cognitive capacity and underlying mental health issues, social status and the anticipated degree of social support, nutritional status and the presence of any geriatric syndromes, which is a term encompassing many issues of the elderly including falls, delirium and dementia, osteoporosis and polypharmacy, amongst others (210). In HNC populations, the CGA may be more adept at uncovering impairments beyond that of the multi-disciplinary team; it has also been associated with the length of post-operative inpatient stays and the rates of surgical complications and successful completion of radiotherapy (211–214). Deficits in some of the CGA domains may predict more significant declines in global QoL (215) or other HRQL domains (214) after treatment in HNC patients. The implementation and use of CGA in routine clinical practice is, however, resource intensive and while a comprehensive assessment of every older patient being considered for curative-intent HNC treatment would no doubt lead to many opportunities for pre-treatment optimization, how best to incorporate a CGA into routine practice would need considerable resource and multi-disciplinary investment at a local or institutional level.

## Limitations

This narrative review provides an overview of findings from studies investigating health and wellbeing outcomes and unmet needs in older and elderly patients with HNC and HNSCC and contrasts them to outcomes reported in younger HNC patients. Of the available studies, there are several limitations hampering the ability to draw firm conclusions. Most notably the data is hindered by a lack of consensus in defining the threshold for an older HNC patient. These studies which largely included patients treated with radical treatment also represent the outcomes for a biased population of robust older patients and their outcomes are likely not generalizable to all older patients with HNC, particularly those who may be more frail. It is also worth considering that these studies did not include detailed information about the treatments received, and whether older patients may have undergone reduced intensity treatment to accommodate their age, such as alterations in surgical fields, radiation volumes or chemotherapy dosing. The available studies also vary widely in the different PROs used, the methodologies used in their application and the statistical methods with which they have been analyzed and interpreted. Future studies and high-quality research will hopefully add to our understanding and avoid some of the aforementioned limitations. Well-defined secondary analyses from prospective phase II or III studies where older patients are included would be a welcome addition to the literature. In this review, where possible we have focused on those studies including only HNSCC, however, this was not always possible particularly with the lesser-studied psychosocial distress outcomes. However, despite their histological similarities, HNSCC encompasses a diverse range of tumors, including tumors with different etiology (HPV or smoking-related), primary sites and treatment, factors which may all impart different HRQL and psychosocial challenges for individual HNC patients. Interpretation outcomes from the various studies included in this review was frequently hampered by a reliance on statistical inferences rather than focusing on differences that would be clinically relevant, a theme which echoes throughout most of the PRO literature. Future studies of age-related changes would benefit from taking into account what is already known about changes over the life course in the general population. This would further our understanding of cancer- and treatment-related issues in younger and older patients. Future research exploring variations in age-related outcomes in HNC populations will need to focus on these challenges to draw more meaningful conclusions for our patients.

## Conclusions and Future Directions

While there are many limitations to the available data this review should be reassuring when considering intensive treatment in the older HNC and HNSCC population. In synthesizing the varied health and wellbeing outcomes included in this report, when undergoing curative intent protocols, older patients have tended to demonstrate more resilience in HRQL and psychosocial outcomes when compared to their younger counterparts, with the caveat the older patients included in most of these studies were considered robust enough to treat with radical treatment. While refining optimal survivorship models will remain across the continuum of patients presenting with HNC, these findings suggests that we should be in fact more aware of the support that younger HNC patients may need across multiple HRQL and psychosocial distress domains following HNC treatment, despite their perceived physical robustness. Future research efforts in reporting age-related variations in HRQL, psychosocial distress and unmet needs will need to work towards harmonizing the definition of the older HNC patient, narrow the research question in more homogenous HNC populations and investigate novel approaches to measure biological rather than chronological age.

## Author Contributions

LM and CH designed the concept of the submitted manuscript, were responsible for data acquisition and wrote the first draft. All authors (LM, CH, KG, and DR) were involved in the interpretation of the data included and in drafting and revising the first draft. All authors have provided approval for publication of the included content.

## Conflict of Interest

The authors declare that the research was conducted in the absence of any commercial or financial relationships that could be construed as a potential conflict of interest.

## Publisher’s Note

All claims expressed in this article are solely those of the authors and do not necessarily represent those of their affiliated organizations, or those of the publisher, the editors and the reviewers. Any product that may be evaluated in this article, or claim that may be made by its manufacturer, is not guaranteed or endorsed by the publisher.
